# Microglia Responses to Pro-inflammatory Stimuli (LPS, IFNγ+TNFα) and Reprogramming by Resolving Cytokines (IL-4, IL-10)

**DOI:** 10.3389/fncel.2018.00215

**Published:** 2018-07-24

**Authors:** Starlee Lively, Lyanne C. Schlichter

**Affiliations:** ^1^Division of Genetics & Development, Krembil Research Institute, University Health Network, Toronto, ON, Canada; ^2^Department of Physiology, University of Toronto, Toronto, ON, Canada

**Keywords:** microglial activation, pro-inflammatory stimuli, LPS, IFNγ plus TNFα, transcription profiling, resolving cytokines

## Abstract

Microglia respond to CNS injuries and diseases with complex reactions, often called “activation.” A pro-inflammatory phenotype (also called classical or M1 activation) lies at one extreme of the reactivity spectrum. There were several motivations for this study. First, bacterial endotoxin (lipopolysaccharide, LPS) is the most commonly used pro-inflammatory stimulus for microglia, both *in vitro* and *in vivo*; however, pro-inflammatory cytokines (e.g., IFNγ, TNFα) rather than LPS will be encountered with sterile CNS damage and disease. We lack direct comparisons of responses between LPS and such cytokines. Second, while transcriptional profiling is providing substantial data on microglial responses to LPS, these studies mainly use mouse cells and models, and there is increasing evidence that responses of rat microglia can differ. Third, the cytokine milieu is dynamic after acute CNS damage, and an important question in microglial biology is: How malleable are their responses? There are very few studies of effects of resolving cytokines, particularly for rat microglia, and much of the work has focused on pro-inflammatory outcomes. Here, we first exposed primary rat microglia to LPS or to IFNγ+TNFα (I+T) and compared hallmark functional (nitric oxide production, migration) and molecular responses (almost 100 genes), including surface receptors that can be considered part of the sensome. Protein changes for exemplary molecules were also quantified: ARG1, CD206/MRC1, COX-2, iNOS, and PYK2. Despite some similarities, there were notable differences in responses to LPS and I+T. For instance, LPS often evoked higher pro-inflammatory gene expression and also increased several anti-inflammatory genes. Second, we compared the ability of two anti-inflammatory, resolving cytokines (IL-4, IL-10), to counteract responses to LPS and I+T. IL-4 was more effective after I+T than after LPS, and IL-10 was surprisingly ineffective after either stimulus. These results should prove useful in modeling microglial reactivity *in vitro*; and comparing transcriptional responses to sterile CNS inflammation *in vivo*.

## Introduction

Central nervous system (CNS) injury and disease states are marked by complex reactions of microglia, the brain’s endogenous immune cells. Microglia can rapidly respond to environmental cues (e.g., stranger and danger signals) by transitioning from a surveillance mode to various states of activation or reactivity. The most well-studied reactive response to infection or damage is a multi-faceted pro-inflammatory phenotype accompanied by release of cytokines and reactive oxygen and nitrogen species that can potentially exacerbate the damage (Mantovani et al., 2004; Cherry et al., 2014; Orihuela et al., 2016). One potential limitation is that most *in vitro* studies of microglial pro-inflammatory states have applied the bacterial cell wall component, lipopolysaccharide (LPS) to evoke a state that has been called “classical” or M1 activation by analogy to macrophage reactions (Colton, 2009; Kettenmann et al., 2011; Hanisch, 2013; Cherry et al., 2014; Franco and Fernandez-Suarez, 2015). Indeed, responses to LPS have been well characterized *in vitro* and *in vivo* (reviewed in Perry and Andersson, 1992; Lund et al., 2006; Wang et al., 2006; Hoogland et al., 2015). Because most CNS injuries occur without a bacterial infection in the brain; other pro-inflammatory stimuli will be more relevant. This study was initially motivated by the need for more information concerning responses of microglia to physiologically relevant endogenous stimuli, such as cytokines.

In selecting pro-inflammatory stimuli to compare with LPS, we chose to examine microglial responses to interferon-γ (IFNγ) and tumor necrosis factor-α (TNFα), for several reasons. (1) IFNγ and TNFα bind to receptors on microglia and other brain cells (reviewed in Benveniste and Benos, 1995), and several earlier studies used IFNγ and TNFα as a pro-inflammatory stimulus for microglia *in vitro* (Spanaus et al., 1998; Suk et al., 2001; Mir et al., 2008). (2) Both cytokines are elevated within the CNS in numerous pathologies and damage models, including stroke, trauma, spinal cord injury, perforant-path axotomy, and in multiple sclerosis (MS) and other neurodegenerative disorders (Benveniste and Benos, 1995; Elliott, 2001; Li et al., 2001; Tarkowski et al., 2003; Yamamoto et al., 2007; Barcia et al., 2011; Woodcock and Morganti-Kossmann, 2013; Kroner et al., 2014). Moreover, chronic elevations of IFNγ and TNFα are involved in initiating and/or maintaining glial activation in a macaque model of Parkinson’s Disease (Barcia et al., 2011). (3) Our work and others using rat models of ischemic and hemorrhagic stroke have shown early rises in both cytokines within the lesioned sites (Li et al., 2001; Wasserman et al., 2007; Sieber et al., 2011; Lively et al., 2016), and we found a crucial contribution of TNFα to neuron killing in a model of the stroke penumbra (Kaushal and Schlichter, 2008). (4) Our recent *in vitro* studies using IFNγ and TNFα (I+T) have alerted us to numerous functional changes that could have important consequences for microglial contributions to neuro-inflammation. These include changes in myelin phagocytosis and production of reactive oxygen and nitrogen species (Siddiqui et al., 2016; Lam et al., 2017); in migratory capacity (Lam et al., 2017), and in levels of several potassium channels that are possible therapeutic targets (Siddiqui et al., 2016; Lam et al., 2017).

The second part of this study was motivated by the burgeoning information concerning time-dependent changes in the chemical milieu in acutely damaged brain tissue, which are expected to affect microglial phenotypes and functions (Crotti and Ransohoff, 2016; Morganti et al., 2016). We previously observed concurrent elevation of pro- and anti-inflammatory mediators (Wasserman et al., 2007; Lively and Schlichter, 2012), and we were intrigued by reports that interleukin (IL)-4 (Li et al., 2001; Zhao et al., 2015) and IL-10 (Sieber et al., 2011) transcript levels peaked within 6 h after stroke. Our work on a rat model of intracerebral hemorrhage also showed elevated IL-10 mRNA at 6 h, and it remained elevated as long as 6 days later (Wasserman et al., 2007). IL-4 and IL-10 are well-known anti-inflammatory cytokines that induce states *in vitro* that have variously been called alternative activation (M2a) and acquired deactivation (M2c), respectively (Colton, 2009; Hanisch, 2013; Cherry et al., 2014). Both cytokines are commonly used on microglia *in vitro* and there is considerable information about their receptors and signaling pathways, and about molecular changes they evoke (reviewed in Gadani et al., 2012; Lobo-Silva et al., 2016). In our own work on rat microglia, IL-4 and IL-10 increased migration and invasion (Lively and Schlichter, 2013; Siddiqui et al., 2014) but the two cytokines also showed divergent effects. For instance, only IL-10 increased myelin phagocytosis (Siddiqui et al., 2016) and podosome expression (Siddiqui et al., 2014); and only IL-4 increased Kv1.3 channel expression and current (Lam et al., 2017). Very little is known about competition and consequences of sequential exposure of microglia to pro- versus anti-inflammatory stimuli. For rat microglia, two *in vitro* studies found that adding IL-4 before (Kitamura et al., 2000) or at the same time as LPS (Ledeboer et al., 2000) decreased induction of inducible nitric oxide synthase (iNOS), IL-6 and TNF-α. For mouse microglia, adding IL-4 after LPS decreased expression of iNOS and cyclooxygenase 2 (COX-2) and increased mannose receptor (CD206/MRC1) and arginase I (ARG1) (Fenn et al., 2012; Chhor et al., 2013). We recently found that subsequent addition of IL-4 or IL-10 partially reversed effects of I+T treatment on myelin phagocytosis and the consequent respiratory burst and expression of inflammatory markers (Siddiqui et al., 2016). Here, we examined cytokine competition by adding either IL-4 or IL-10 shortly after I+T treatment.

First, we compared responses of primary rat microglia to LPS and I+T using targeted transcription profiling of a wide range of inflammation-related molecules (pro- and anti-inflammatory mediators, receptors, immunomodulators, ion channels), and quantified changes in several selected proteins [ARG1, CD206, COX-2, iNOS, protein tyrosine kinase 2 beta (PYK2)] and functional responses [nitric oxide (NO) production, migration]. We found that LPS evoked a more robust pro-inflammatory response, but also elevated a wider range of anti-inflammatory mediators than I+T. Second, we compared the abilities of IL-4 and IL-10 to interfere with outcomes of the two pro-inflammatory treatments. Both cytokines interfered with I+T-evoked responses more effectively than LPS-evoked responses, and IL-4 was more effective than IL-10. Together, our findings demonstrate differential effects of pro- and anti-inflammatory stimuli on microglial molecular phenotypes and functions. Such differences will be important to consider when assessing inflammatory profiles *in vitro* and in examining sterile and non-sterile forms of CNS damage.

## Materials and Methods

### Isolation and Stimulation of Primary Rat Microglia

All procedures on animals were in accordance with the Canadian Council on Animal Care and approved by the University Health Network Animal Care Committee (Animal Use Protocol #914).

Microglia were isolated from 1 to 2-day old Sprague-Dawley rat pups (Charles River, St.-Constant, PQ, Canada) using standard operating protocols that we find yield essentially pure microglia, as determined by labeling with tomato lectin, isolectin B4, or antibodies against Iba1 or CD11b (Cayabyab et al., 2000; Khanna et al., 2001; Ducharme et al., 2007; Ohana et al., 2009; Schlichter et al., 2010; Sivagnanam et al., 2010; Lively and Schlichter, 2013; Lam and Schlichter, 2015; Siddiqui et al., 2016; Lively et al., 2018). Anti-CD11b staining of the present cultures is shown in Supplementary Figure 1A. We find that these microglia have very low expression of many inflammatory mediators that are characteristic of more activated cells (e.g., Sivagnanam et al., 2010; Liu et al., 2013; Lam and Schlichter, 2015; Siddiqui et al., 2016; Lively et al., 2018). Of course, they are not “quiescent.” For instance, as appropriate for neonatal microglia that are involved in refining the brain architecture, many are unipolar with a large lamellum and a uropod and are highly migratory (Lively and Schlichter, 2012, 2013; Siddiqui et al., 2012, 2016; Vincent et al., 2012; Lam et al., 2017; Lively et al., 2018). The cerebellum was removed and the remaining brain tissue was minced in cold Minimal Essential Medium (MEM; ThermoFisher Scientific, **RRID**:SCR_008452; Cat# 11095080), strained and centrifuged at 300 × *g* for 10 min. The pellet was re-suspended in MEM containing 10% fetal bovine serum (FBS; Wisent, Saint-Jean-Baptiste, QC, Canada; Cat# 080-150) and 0.05 mg/mL gentamycin (ThermoFisher Scientific; Cat# 15710072), and the cells were seeded in tissue culture flasks and incubated at 37°C and 5% CO_2_. After 48 h, the medium was changed and the cells were cultured for 5–6 days. Microglia were removed from the astrocyte bed by gently shaking the flasks for 3–4 h on an orbital shaker (70 rpm; 37°C, 5% CO_2_), then centrifuging at 300 × *g* for 10 min. The microglia pellet was resuspended in MEM containing 2% heat-inactivated FBS and 0.05 mg/mL gentamycin. Microglia were seeded on coverslips at ∼3 × 10^4^ cells/Transwell insert for migration assays, ∼6 × 10^4^ cells/15 mm coverslip for fluorescence microscopy and NO production, and >10^5^ cells/coverslip for mRNA isolation. After plating, the microglia were incubated for 24 h, at which time they were healthy looking (see images in Results).

We chose cytokine concentrations and time points for examining outcomes based on previous studies from our laboratory and others that reported effects on microglial responses. Many studies have examined microglial responses 24 h after LPS stimulation; thus, we compared LPS with IFNγ+TNFα at 24 h. A wide range of LPS concentrations has been used (10 ng/mL–2 μg/mL). Low concentrations (<100 ng/mL) reliably alter microglial morphology and functions (Visentin et al., 1995; Ledeboer et al., 2000; Zujovic et al., 2000; Lieb et al., 2003; Qian et al., 2006; Kaushal et al., 2007; Sivagnanam et al., 2010; Lively and Schlichter, 2013) but high concentrations decrease viability of rat microglia (von Zahn et al., 1997; Sivagnanam et al., 2010). In our experience, 10 ng/mL of LPS derived from *E. coli* K-235 bacteria (Sigma-Aldrich; Oakville, ON, Canada Cat # L2018), as used in this study, is optimal for inducing pro-inflammatory responses without toxicity (Sivagnanam et al., 2010; Lively and Schlichter, 2013). Concentrations of the other cytokines were based on previous studies reporting microglia responses. We used 20 ng/mL IFNγ (R&D Systems Inc., **RRID**:SCR_006140; Cat# 585-IF; Hu et al., 2012; Siddiqui et al., 2016), 50 ng/mL TNFα (R&D Systems Inc., Cat# 510-RT; Kuno et al., 2005; Siddiqui et al., 2016); 20 ng/mL IL-4 (R&D Systems Inc., Cat # 504-RL; Liu et al., 2010; Hu et al., 2012; Girard et al., 2013; Lively and Schlichter, 2013), and 20 ng/mL IL-10 (R&D Systems Inc., Cat# 522-RL; Wirjatijasa et al., 2002; Qian et al., 2006; Liu et al., 2010; Lam and Schlichter, 2015).

To assess the ability of resolving cytokines to interfere with the pro-inflammatory program, we added IL-4 or IL-10 at 2 h after adding LPS or I+T. The idea was to allow receptor-mediated signaling to be initiated by the cognate membrane receptors (TNFR’s, IFNγR’s, TLR4/MyD88), and then determine if IL-4 or IL-10 could interfere with their responses. By 2 h after adding LPS to cultured rat microglia, increased transcript levels of hallmark pro-inflammatory mediators have been observed (Kitamura et al., 2000). Another reason we were interested in this form of competition (compared with more delayed cytokine treatments) was that, in stroke studies, increases in IL-4 and IL-10 can temporally and spatially overlap with increases in pro-inflammatory mediators (see “Introduction”). Stock solutions were made in sterile phosphate buffered saline (PBS; Wisent; Cat# 311-010-CL) with 0.3% bovine serum albumin (BSA; Bioshop, Burlington, ON, Canada; Cat# ALB001) and stored at –20°C. Fresh aliquots were used for each microglia culture.

### Transcriptional Analysis

Microglia were seeded at 5 × 10^5^ cells/coverslip in a 12-well culture plate and allowed to settle for 1–2 days (37°C, 5% CO_2_) before stimulation for 6 or 24 h. Total RNA was extracted using TRIzol reagent (ThermoFisher Scientific; Cat# 15596018) and RNeasy Mini Kits (QIAGEN, Mississauga, ON, Canada; Cat# 74104) and samples were stored at –80°C. The nCounter gene expression assay (NanoString) was used, as before (Ferreira et al., 2014; Siddiqui et al., 2016; Lam et al., 2017) to analyze transcript levels of numerous genes in each RNA sample. For each sample, 200 ng of extracted RNA was sent to the Princess Margaret Genomics Centre^1^(TO, Canada), where the sample purity was assessed (using Nanodrop 1000) and the assay conducted (hybridization, detection, scanning). Samples were obtained from 6 to 7 individual microglia cultures at 24 h for data in the main Tables and Figures. For the Supplementary data at 6 h, 3–5 cultures were used.

NanoString nCounter^TM^ technologies designed the code set, which consists of capture and reporter probes (Supplementary Tables 1, 2). Raw data were analyzed using nSolver^TM^ Analysis Software (ver3.0; **RRID**:SCR_00342). To standardize the assay, negative reporter probes were used for background subtraction and positive probes for irrelevant control genes were used to assess hybridization efficiency, detection range, and to calculate a scaling factor that was applied to all mRNA counts in each sample. Finally, a reference gene scaling factor was calculated in the same manner using the housekeeping genes, *Gusb* (glucuronidase beta), *Rpl32* (ribosomal protein L32), *Hprt1* (hypoxanthine phosphoribosyltransferase 1). The 6 h assay used *Hprt1*, *Sdha* (succinate dehydrogenase complex flavoprotein subunit A) and *Ywhaz* (tyrosine 3-monooxygenase/tryptophan 5-monooxygenase activation protein, zeta). Normalized data were log2-transformed for further statistical analysis. In the figures and tables, control data are shown as normalized mRNA counts to highlight the magnitude differences in transcript levels from gene to gene.

### Western Blot Analysis

Western blotting was used to determine whether observed mRNA changes correlated in time with protein changes, as before (Lam et al., 2017; Lively et al., 2018). Microglia were seeded on 25 mm coverslips (Fisher Scientific, Ottawa, ON, Canada; Cat# 12-545-86) in 35 mm culture dishes at 1–3 × 10^6^ cells (5–6 independent cell cultures). After incubating overnight, cells were treated with LPS or I+T for 24 h (single stimulation) or for 2 h followed by addition of IL-4 or IL-10 for 22 h (sequential stimulation). Cells were harvested by briefly washing with PBS and lysing for 30 min in ice-cold RIPA buffer that contained a mammalian protease inhibitor cocktail (Sigma-Aldrich; Cat# P3840). Insoluble material was removed by centrifuging and discarding the pellet. The protein concentration in the supernatant was determined with a Pierce^TM^ BCA protein assay (ThermoFisher Scientific; Cat# 23225), and lysates were stored at –80°C. Just before use, proteins were denatured (100°C for 5 min in a dry-bath incubator) in NuPage LDS sample buffer (ThermoFisher Scientific; Cat# NP0007) containing 5% 2-β-mercaptoethanol. Samples were loaded at 10 μg protein/lane on 8 or 12% acrylamide gels and subjected SDS-PAGE for 1.5–2 h at 80 mV (stacking gel) and 120 mV (resolving gel). Proteins were then transferred to a PVDF membrane and blocked for 2–3 h in 5% non-fat dry milk in Tris-Tween buffered saline (TTBS).

Protein levels were measured for ARG1, CD206, COX-2, iNOS, and PYK2. Primary antibodies (incubated overnight at 4°C) were diluted in TTBS with 1% BSA, as follows: rabbit anti-liver ARG (1:2000; Abcam, Cat# ab91279, **RRID**:AB_10674215), rabbit anti-CD206 (1:2000; Abcam, Cat# ab64693, **RRID**:AB_1523910), rabbit anti-COX-2 (1:1000; Abcam Cat# ab15191, **RRID**:AB_2085144), mouse anti-iNOS (Abcam Cat# ab49999, **RRID**:AB_881438), and rabbit anti-PYK2 (Abcam Cat# ab32571, **RRID**:AB_777566). After washing in 1% BSA-TTBS (4 × 10 min), membranes were incubated at room temperature for 1 h in horseradish peroxidase-labeled secondary antibodies (1:3000; Cedarlane, Burlington, ON, Canada, **RRID**:SCR_004462; anti-rabbit IgG: Cat# CLCC42007; anti-mouse IgG: Cat # CLCC30207) in 1% BSA-TTBS. After washing (6×, 5 min each), membranes were treated for 2 min with GE Healthcare ECL^TM^ Start Western Blotting Detection Reagent (Sigma-Aldrich; Cat# GERPN3243). Protein band intensities were captured using the ChemiDocTM XRS System (Bio-Rad).

To compare changes in protein levels, total protein normalization was used, as before (Lam et al., 2017; Lively et al., 2018). Membranes were stained for 1 min with 0.1% Coomassie Brilliant Blue G (Sigma-Aldrich; Cat# B8522), de-stained for 2 min in acetic acid/methanol/water (1:5:4), air-dried, and imaged with a ChemiDocTM XRS System. Image Lab (ver.5.2.1, **RRID**:SCR_014210) was used to identify gel lanes and bands of interest, and to subtract the background and determine signal intensities of identified bands. Bands of interest were then normalized to the total Coomassie blue staining intensity of a given lane, and then expressed as fold-changes relative to unstimulated (control) cells. Supplementary Figures 1B, 2 show uncropped images of representative blots used for quantification.

### Microglia Staining

After stimulating microglia for 24 h, the cells were quickly washed in PBS and fixed at room temperature for 10 min in 4% paraformaldehyde (PFA; Electron Microscopy Sciences, Hatfield, PA, United States; Cat# 15710). Fixed cells were quickly washed three times in PBS and permeabilized with 0.2% Triton X-100 for 5 min. To examine morphology, filamentous (F) actin was visualized by incubating permeabilized cells with Acti-stain 488 phalloidin (1:100 in PBS; Cytoskeleton Inc., **RRID**:SCR_013532; Cat# PHDG1-A) for 1 h at room temperature, and counterstained with the nuclear dye, 4′,6-diamidino-2-phenylindole (DAPI; 1:3000 in PBS; Sigma-Aldrich; Cat# D9542) for 5 min. Coverslips were then mounted on glass slides in DAKO mounting medium (Agilent-Dako, **RRID**:SCR_013530; Cat# S302380-2) and stored in the dark at 4°C. Images were acquired using a Zeiss 880 confocal microscope (model LSM880; Zeiss, Oberkochen, Germany) and captured using Zen software (version 2.3 SPI; Zeiss, Toronto, ON, Canada).

### Migration Assay

Microglia were seeded on Transwell inserts (VWR; Cat# CA 62406-198) bearing 8 μm-diameter holes, in a solution of 500 μL MEM with 2% FBS, as before (Lively and Schlichter, 2013; Siddiqui et al., 2014; Lam and Schlichter, 2015). The cells were allowed to settle for 30 min (37°C, 5% CO_2_), and then 500 μL MEM with 2% FBS was added to the lower wells, and cells were stimulated as described above. After 24 h, the cell-bearing filters were fixed in 4% PFA for 10 min, quickly rinsed (3× with PBS), and the inner side of the membrane was swirled with a Q-tip to remove any cells that had not migrated. The filters were stained with 0.3% crystal violet for 1 min and then rinsed with PBS. Cells that had migrated to the underside of the filter were counted (sum of 5 random fields/filter) at 20 × magnification using an Olympus CK2 inverted microscope (Olympus, Tokyo, Japan). Cell counts were normalized to the unstimulated (control) group.

### Nitric Oxide Production

The colorimetric Griess assay was used to measure nitrite, which is proportional to NO production. Two hundred μL of supernatant from each microglia sample was added to a well in a 96-well plate that contained 25 μl of 1% sulfanilic acid (Sigma-Aldrich; Cat#86090). Then, 25 μl of 0.1% *N*-(1-naphthyl)ethylene diamine dihydrochloride (Sigma-Aldrich; Cat#222488) was added and the plate was stored for 30 min at room temperature in the dark to allow the reaction to occur. The colorimetric change was quantified using a multi-label plate counter (Victor^3^ 1420, Perkin Elmer, Woodbridge, ON, Canada) with absorbance set to 570 nm. The nitrite concentration in each sample was interpolated from a standard curve generated from a series of NaNO_2_ samples of known concentration.

### Statistics

All graphical data are presented as mean ± SEM for the number of biological replicates indicated, and statistical significance was analyzed using GraphPad ver 6.01 (**RRID**:SCR_002798). Western blotting, migration and invasion data were analyzed using a one-way analysis of variance (ANOVA) with Tukey’s *post-hoc* test. For NanoString data, after normalizing to housekeeping genes, log2 count values were analyzed by one-way ANOVA with Fisher’s LSD test to identify expression changes induced by LPS or I+T. For sequential stimulation with IL-4 or IL-10, counts were expressed as fold-change and analyzed with a two-way ANOVA with Fisher’s LSD test. The *p* values for each gene were then adjusted for multiple comparisons using a 5% false discovery rate correction (Benjamini and Yekutieli, 2001) in the program R (version 3.3.1; R Project for Statistical Computing, **RRID**:SCR_001905). Results were considered significantly different if *p* < 0.05.

## Results

### Verifying That Primary Rat Microglia Respond to LPS and IFNγ+TNFα

**FIGURE 1:**
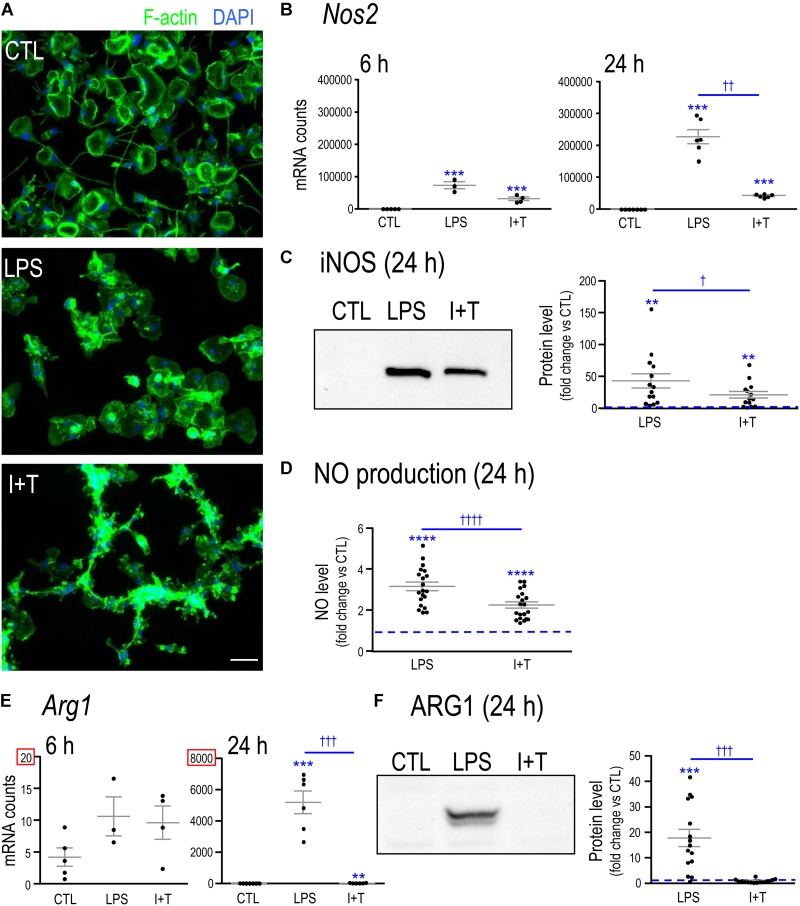
Verifying that primary rat microglia responded to LPS and to IFNγ+TNFα. **(A)** Representative fluorescence images of primary rat microglia (unstimulated, CTL) after 24 h treatment with LPS or a combination of IFNγ + TNFα (I+T). Fixed microglia were stained for F actin (phalloidin; green) and nuclei were labeled with DAPI (blue). Scale bar, 50 μm. **(B)** *Nos2* mRNA levels were measured by Nanostring and are expressed as mRNA counts/200 ng RNA sample (6 h: 3–5 individual cultures; 24 h: 6–7 individual cultures). **(C)** iNOS protein levels at 24 h. Left: Representative Western blot. Blots were analyzed and normalized to total Coomassie blue staining. Right: Fold-changes with respect to controls (dashed line) were plotted for separate replicates from different cultures (14 individual cultures). **(D)** Cumulative nitric oxide (NO) production for the 24 h period after stimulation (20 individual cultures). **(E)** *Arg1* mRNA levels were measured and analyzed as in Panel B. **(F)** Arg1 protein levels at 24 h. Western blots were analyzed as in Panel C (15 individual cultures). For all graphs, values are plotted as mean ± SEM, and differences are indicated with respect to control microglia (^∗^) and between stimuli (†). One symbol of either type indicates *p* < 0.05; two symbols, *p* < 0.01; three symbols, *p* < 0.001; four symbols, *p* < 0.0001.

To show that the microglia had responded to LPS and I+T, we first assessed changes in their morphology and in the expression of two molecules, iNOS and ARG1, that are routinely used as hallmarks of pro- and anti-inflammatory states, respectively. Unstimulated microglia were predominantly unipolar with a large F-actin-rich ring in each lamellum (**Figure 1A**). A 24 h exposure to LPS caused most microglia to retract their processes, and become rounded or amoeboid, as previously shown (Lively and Schlichter, 2013). I+T treatment produced a different morphology, with mainly round or small cells bearing multiple short processes and in chain-like groupings. Production of NO is commonly used to indicate a microglial response to LPS; and is due to up-regulation of *Nos2* mRNA and iNOS protein. Here, we found that LPS up-regulated *Nos2* expression as early as 6 h and by 24 h the level was very high (**Figure 1B**). I+T treatment induced smaller increases. iNOS protein (**Figure 1C**) and NO production (**Figure 1D**) were increased by both stimuli at 24 h but to slightly higher levels by LPS. Of particular note was the dramatic up-regulation of *Arg1* mRNA (**Figure 1E**) and ARG1 protein 24 h following LPS treatment (**Figure 1F**). In contrast, the small increase in *Arg1* mRNA in response to I+T did not result in a detectable protein change.

### Similarities and Differences in Responses to LPS Versus IFNγ+TNFα

#### Pro- And Anti-inflammatory Genes and Receptors

##### Unstimulated

We have previously shown that unstimulated rat microglia were in a relatively resting state (Sivagnanam et al., 2010; Lively and Schlichter, 2013; Siddiqui et al., 2016; Lam et al., 2017), and this was corroborated in the present study. That is, **Tables 1**, **2** show low baseline transcript expression (arbitrary cutoff, <500 mRNA counts/200 ng RNA) of common pro-inflammatory [*Casp1* (*Caspase-1*/IL-1-converting enzyme/ICE), Ifnγ, *Il6*, *Nos2, Ptsg2* (COX-2), *Tnf*] and anti-inflammatory molecules [*Arg1, Ccl22*, *Cd163*, *Chi3l3* (YM1), *Il4*, *Il10*, *Retnla* (FIZZ1)].

**Table 1:** Comparing effects of LPS and I+T on transcript levels of pro-inflammatory mediators.

	mRNA counts	Fold change with respect to Control	
		
Gene	Control	LPS	I+T
*C1r*	59 ± 40	4.15 ± 2.39^↑↑^	5.54 ± 3.35^↑↑↑^
*C5ar1*	2825 ± 665	6.86 ± 1.3^↑↑↑***^	0.25 ± 0.07^↓↓↓^
*Casp1* (ICE)	475 ± 112	2.80 ± 0.72^↑↑↑**^	1.55 ± 0.10^↑^
*Ccl3*	3667 ± 823	71.68 ± 16.04^↑↑↑***^	0.52 ± 0.12^↓↓^
*Ifng*	4 ± 3	4.17 ± 3.60^↑^	1.50 ± 1.39
*Ifngr1*	5054 ± 717	1.71 ± 0.71	1.26 ± 0.26
*Ifngr2*	18 ± 7	2.11 ± 0.50^↑↑↑^	1.52 ± 0.22^↑^
*Il1b*	1011 ± 607	192.60 ± 31.04^↑↑↑***^	1.24 ± 0.33
*Il1r1*	7 ± 3	14.79 ± 5.41^↑↑↑***^	1.72 ± 0.48
*Il1r2*	7 ± 4	7.79 ± 5.20^↑↑↑*^	1.80 ± 0.75
*Il6*	5 ± 4	2910.45 ± 649.57^↑↑↑***^	4.17 ± 2.23^↑↑↑^
*Nos2* (iNOS)	41 ± 42	5479.50 ± 1313.28^↑↑↑**^	1030.13 ± 125.70^↑↑↑^
*Ptgs2* (COX-2)	13 ± 10	1471.12 ± 710.39^↑↑↑***^	28.66 ± 17.08^↑↑↑^
*Ptk2b* (PYK2)	875 ± 182	6.28 ± 0.97^↑↑↑^	11.75 ± 0.43^↑↑↑***^
*Tnf* (TNFα)	325 ± 127	8.15 ± 1.60^↑↑↑*^	4.20 ± 0.88^↑↑↑^
*Tnfrsf1a* (TNFR1)	657 ± 67	3.38 ± 0.77^↑↑↑^	3.49 ± 0.40^↑↑↑^
*Tnfrsf1b* (TNFR2)	1126 ± 139	22.91 ± 4.37^↑↑↑***^	2.71 ± 0.40^↑↑↑^


*Rat microglia were stimulated for 24 h with LPS or a combination of IFNγ + TNFα (I+T). For clarity, protein names are included in parentheses for some common genes. Basal mRNA levels in unstimulated (control) cells are expressed as mean counts/200 ng RNA sample ± SD (*n* = 6–7 individual microglia cultures). Fold changes were used to assess effects of LPS or I+T on each gene. Arrows indicate statistically significant increases (↑) or decreases (↓) relative to control cells. Asterisks (^∗^) indicate differences between LPS and I+T. One symbol of either type indicates *p* < 0.05; two, *p* < 0.01; three, *p* < 0.001.*

**Table 2:** Transcript expression of anti-inflammatory genes and receptors.

	mRNA counts	Fold change with respect to Control	
		
Gene	Control	LPS	I+T
*Arg1*	4 ± 3	1216.02 ± 419.03^↑↑↑***^	4.73 ± 3.00^↑↑^
*Ccl22*	5 ± 4	81.39 ± 42.61^↑↑↑***^	3.36 ± 0.70^↑↑^
*Cd163*	4 ± 2	8.10 ± 3.85^↑↑↑**^	1.69 ± 1.06
*Chi3l3* (YM1)	5 ± 3	3.46 ± 1.03^↑↑^	1.57 ± 0.84
*Il1rn* (IL-1RA)	2625 ± 985	2.42 ± 1.58	3.37 ± 1.17^↑↑^
*Il4*	6 ± 3	4.58 ± 3.40^↑*^	1.04 ± 0.45
*Il4r*	374 ± 24	10.16 ± 3.19^↑↑↑^	7.38 ± 0.99^↑↑↑^
*Il10*	9 ± 7	36.88 ± 10.75^↑↑↑***^	0.24 ± 0.16
*Il10ra*	561 ± 64	2.09 ± 0.36^↑↑↑^	4.10 ± 0.59^↑↑↑***^
*Il10rb*	999 ± 103	3.18 ± 0.59^↑↑↑***^	2.00 ± 0.13^↑↑↑^
*Il13ra1*	350 ± 45	6.39 ± 1.36^↑↑↑***^	2.57 ± 0.31^↑↑↑^
*Mrc1* (CD206)	1110 ± 570	1.13 ± 0.40	0.03 ± 0.03^↓↓↓***^
*Myc*	423 ± 56	1.05 ± 0.33	0.24 ± 0.11^↓↓↓***^
*Pparg*	502 ± 217	0.14 ± 0.05^↓↓↓^	0.06 ± 0.03^↓↓↓*^
*Retnla* (FIZZ1)	3 ± 2	3.80 ± 1.29^↑↑^	1.60 ± 1.01
*Tgfb1*	10358 ± 760	1.18 ± 0.16	0.51 ± 0.07^↓↓↓***^
*Tgfbr1*	2445 ± 310	0.73 ± 0.17^↓***^	1.21 ± 0.21
*Tgfbr2*	769 ± 67	3.37 ± 0.61^↑↑↑***^	1.99 ± 0.02^↑↑↑^


*Treatments, data presentation and analysis are as in **Table 1**.*

##### Stimulated

As expected, LPS and I+T both up-regulated several pro-inflammatory molecules. There was increased expression of *C1r*, *Casp1*, *Ifngr2*, *Il6*, *Nos2, Ptgs2*, *Ptk2b* (PYK2), *Tnf*, *Tnfrsf1a* and *Tnfrs1b* (**Table 1**). However, LPS often induced higher levels; i.e., *Casp1*, *Il6*, *Nos2*, *Ptgs2*, *Tnfa*, and *Tnfrs1b*. Moreover, LPS up-regulated several pro-inflammatory mediators that I+T either did not change (*Ifng*, *Il1b*, *Il1r1*, *Il1r2*) or decreased (*C5ar1* and *Ccl3*). In a pilot study at 6 h (Supplementary Table 3), *Il1b* was increased by I+T but less than by LPS. Several pro-inflammatory genes showed early induction at 6 h; e.g., LPS increased *Il6*, *Nos2*, and *Tnfa*, and to a higher degree than did I+T. Some genes showed a delayed response with no change at 6 h (*Casp1*, *Il1r2*). There were some surprising anti-inflammatory responses (**Table 2**). Of the commonly used alternative activation (M2) markers, LPS, but not I+T, increased *Chi3l3* and *Retnla*, suggesting that neither are robust M2 markers in these cells. Moreover, both stimuli increased *Arg1, Ccl22*, *Il4r*, *Il10ra*, *Il10rb*, *Il13ra1*, and *Tgfbr2*, although induction was generally higher in LPS-treated cells, and LPS also increased *Cd163*, *Il4*, and *Il10*. At 6 h, I+T also increased *Il4r* and *Il13ra1* (Supplementary Table 3). Although both LPS and I+T decreased the anti-inflammatory molecule, *Pparg*, at 24 h, many responses differed. LPS (but not I+T) decreased *Tgfbr1*, and I+T (but not LPS) decreased *Mrc1*, *Myc*, and *Tgfb1*.

Overall, both LPS and I+T up-regulated several genes commonly used to delineate a pro-inflammatory state, but LPS up-regulated a wider range of pro-inflammatory mediators. Both stimuli, and especially LPS, increased expression of a mixture of pro- and anti-inflammatory genes. Some of these increases were seen as early as 6 h (e.g., *Ccl22*, *Cd163*, *Il10*, *Il1rn*, *Myc*; Supplementary Table 3), while others were seen only at 24 h; e.g., *Arg1* (LPS and I+T), *Chi3l3*, *Il4r*, and *Il13ra1* (LPS only), and *Tgfb1* (I+T only). Some LPS-induced changes were seen only at 6 h; e.g., *Il1rn* and *Myc*. Overall, the observed mixed responses to two pro-inflammatory stimuli *in vitro* will be important to keep in mind when comparing *in vivo* responses to acute CNS damage.

**FIGURE 2:**
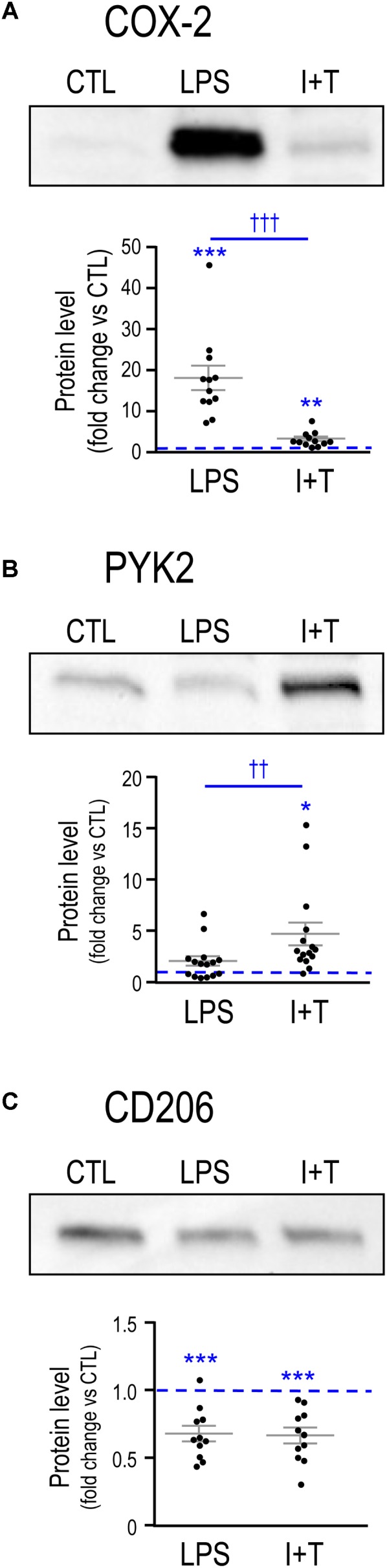
Comparing LPS- and IFNγ+TNFα-induced protein changes for exemplary pro- and anti-inflammatory markers. Representative Western blots and summarized fold-changes in protein levels with respect to control (CTL; dashed line) for the pro-inflammatory markers, COX-2 **(A)** and PYK2 **(B)**, and the anti-inflammatory marker, CD206 **(C)**. Primary rat microglia were harvested 24 h after treatment with LPS or IFNγ+TNFα (I+T). Data are expressed as mean ± SEM (11–15 individual cultures). Differences are indicated with respect to control microglia (^∗^) and between stimuli (†). One symbol indicates *p* < 0.05; two symbols, *p* < 0.01; three symbols, *p* < 0.001.

Next, we examined changes in protein levels for the exemplary molecules, COX-2, PYK2 and CD206 (**Figure 2**) (iNOS and ARG1 protein were described in **Figure 1**). Consistent with the mRNA changes seen at 24 h, LPS induced COX-2 to a much higher level than I+T; while I+T increased PYK2 more than did LPS. For these two molecules, the timing of changes in mRNA and protein were similar. For the anti-inflammatory marker, CD206, both stimuli reduced *Mrc1* mRNA as early as 6 h (Supplementary Table 3) and decreased CD206 protein at 24 h (**Figure 2**); however, *Mrc1* transcript levels had recovered by 24 h after LPS treatment (**Table 2**).

#### Microglia Markers and Immune Modulators

We examined several molecules commonly used to identify activated microglia *in vitro* and *in vivo*: *Aif1* (ionized calcium-binding adapter molecule 1, Iba1), *Cd68* (ED1), *Itgam* (CD11b), as well as several immunomodulatory and signaling molecules, including *Ager* (receptor for advanced glycation end products, RAGE), *Csf1r* (colony-stimulating factor receptor/c-fms), *Cx3cr1* (fraktalkine receptor), *Kdm6b* (Lysine demethylase 6B, JMJD3), *Nfkbia* (nuclear factor of kappa light polypeptide gene enhancer in B-cells inhibitor, alpha/Iκbα), *Nr3c1* (glucocorticoid receptor/GR), *Prkaa1* (5′ adenosine monophosphate-activated protein kinase/AMPK), *Socs1*(Suppressor of cytokine signaling 1), *Socs3*, *Tlr2*, *Tlr4*, *Trem1* (Triggering receptor expressed on myeloid cells 1), *Trem2* and *Tspo* (translocator protein).

##### Unstimulated

As shown in **Table 3**, there was low baseline expression of the receptors, *Ager*, *Ccr2*, *Trem1*, and the signaling molecules, *Kdm6b*, *Prkaa1*, *Socs1*, *Socs3*, and *Tlr4*. Moderate levels (>500 mRNA counts) were seen for *Nfkbia*, the matricellular molecule, *Sparc* (secreted protein acidic and rich in cysteine/osteonectin), and several receptors (*Ccr5*, *Cx3cr1*, *Itgam*, *Nr3c1*, *Tlr2*, *Trem2*, *Tspo*). High baseline expression (>5000 mRNA counts) was seen for *Aif*, *Cd68*, and *Csf1r*.

**Table 3:** Transcript expression of microglia markers and immune modulators.

	mRNA counts	Fold change with respect to Control	
		
Gene	Control	LPS	I+T
*Ager* (RAGE)	2 ± 1	5.99 ± 2.13^↑↑**^	0.84 ± 0.35
*Aif* (Iba1)	16513 ± 2476	3.10 ± 0.50^↑↑↑***^	1.78 ± 0.26^↑↑↑^
*Ccr2*	6 ± 4	7.00 ± 3.82^↑↑↑***^	1.05 ± 0.45
*Ccr5*	1987 ± 471	0.52 ± 0.25^↓^	2.33 ± 0.27^↑↑***^
*Cd68* (ED1)	29185 ± 5270	0.89 ± 0.15	0.64 ± 0.07^↓↓*^
*Csf1r*	18281 ± 1544	0.66 ± 0.18^↓↓***^	1.10 ± 0.07
*Cx3cr1*	754 ± 284	0.12 ± 0.06^↓↓↓^	0.03 ± 0.01^↓↓↓***^
*Itgam* (CD11b)	4080 ± 994	4.17 ± 0.62^↑↑↑***^	1.21 ± 0.12
*Kdm6b* (JMJD3)	213 ± 39	7.87 ± 1.31^↑↑↑***^	2.90 ± 0.24^↑↑↑^
*Nfkbia* (Ikba)	3759 ± 1524	14.55 ± 3.17^↑↑↑**^	5.99 ± 0.40^↑↑↑^
*Nr3c1* (GR)	955 ± 101	2.16 ± 0.38^↑↑↑^	3.87 ± 0.41^↑↑↑***^
*Prkaa1* (AMPK)	389 ± 65	3.48 ± 0.62^↑↑↑***^	1.29 ± 0.18
*Socs1*	11 ± 5	17.90 ± 3.12^↑↑↑^	121.09 ± 34.67^↑↑↑***^
*Socs3*	38 ± 20	226.86 ± 52.43^↑↑↑***^	12.09 ± 4.61^↑↑↑^
*Sparc*	3429 ± 1405	0.26 ± 0.06^↓↓↓^	0.21 ± 0.08^↓↓↓^
*Tlr2*	2206 ± 798	5.30 ± 0.80^↑↑↑***^	1.30 ± 0.32
*Tlr4*	433 ± 75	1.31 ± 0.24	0.66 ± 0.16^↓***^
*Trem1*	131 ± 95	47.70 ± 17.13^↑↑↑***^	1.36 ± 0.36
*Trem2*	3812 ± 713	0.38 ± 0.09^↓↓↓^	0.05 ± 0.02^↓↓↓***^
*Tspo*	1401 ± 616	4.41 ± 1.24^↑↑↑^	3.10 ± 0.54^↑↑↑^


*Treatments, data presentation and analysis are as in **Table 1**.*

##### Stimulated

Many of the genes in this group were increased by LPS (13/20) and the increase was often higher than in response to I+T. The two stimuli shared some effects. Both increased *Aif1*, the transcription factor, *Kdm6b*, the NFκB inhibitor, *Nfkbia*, and *Nr3c1*, *Socs1*, *Socs3*, and *Tspo*. Both treatments decreased *Sparc*, *Cx3cr1*, and *Trem2*. *Cx3cr1* was also decreased at 6 h (Supplementary Table 3). Interestingly, there were some differing or even, opposite responses. Only LPS increased *Ager*, *Ccr2*, *Itgam*, *Tlr2*, and *Trem1* and decreased *Csf1r* and *Ccr5*. Only I+T increased *Ccr5* and decreased *Cd68* and *Tlr4*. Some early, transient responses were detected at 6 h; i.e., increases in *Itgam* and *Tlr2* (I+T only) and a decrease in *Tlr4* (LPS only) (Supplementary Table 3).

#### Genes Related to Microglia Physiological Functions

There is increasing interest in physiological functions of microglia under different activation states. In recent years, we have focused primarily on phagocytosis and the resulting production of reactive oxygen species (ROS) (Sivagnanam et al., 2010; Siddiqui et al., 2016), on migration and invasion through extracellular matrix (ECM; Siddiqui et al., 2012, 2014; Vincent et al., 2012; Ferreira and Schlichter, 2013; Lively and Schlichter, 2013; Ferreira et al., 2014; Lam and Schlichter, 2015), and on Ca^2+^ signaling (see next section). All of the genes examined in this category were altered by one or both pro-inflammatory stimuli (**Table 4**). Several were receptors that promote phagocytosis; e.g., *Axl* (Tyrosine-protein kinase receptor UFO), *Fcgr1a* (CD64), *Fcgr2b* (CD32B), *Fcgr3a* (CD16A), *Msr1* (Macrophage scavenger receptor 1/SR-A/CD204) and *Havcr2* (T-cell immunoglobulin and mucin-domain containing-3/TIM-3); while *Sirpa* (signal regulatory protein alpha) is a negative regulator. Several are involved in NADPH (nicotinamide adenine dinucleotide phosphate)-mediated ROS production: *Cybb* (NADPH oxidase 2/NOX2), *Hvcn1* (Hv1/H^+^ channel), *Ncf1* (p47phox), *Nox1* and *Nox4*. The others were examined because they mediate adhesion and changes in cell morphology [*Itgb2* (Integrin beta chain-2), *Adora1* (adenosine A1 receptor), *Adora2a*] and Ca^2+^ signaling involved in chemotactic migration, phagocytosis and cytokine secretion (*P2rx7*, *P2ry2*, *P2ry6*, *P2ry12*).

**Table 4:** Transcript expression of genes related to microglia physiological functions.

	mRNA counts	Fold change with respect to Control	
		
Gene	Control	LPS	I+T
*Adora1* (A1)	3 ± 3	52.05 ± 10.24^↑↑↑***^	2.56 ± 1.35^↑^
*Adora2a* (A2A)	26 ± 23	54.90 ± 11.80^↑↑↑^	22.56 ± 2.51^↑↑↑^
*Axl*	7177 ± 1053	0.27 ± 0.08^↓↓↓^	0.29 ± 0.08^↓↓↓^
*Cybb* (NOX2)	2216 ± 359	1.12 ± 0.45	2.08 ± 0.29^↑↑**^
*Fcgr1a* (CD64)	3866 ± 1296	3.16 ± 0.95^↑↑↑***^	0.46 ± 0.06^↓↓^
*Fcgr2b* (CD32B)	3480 ± 839	4.74 ± 1.11^↑↑↑***^	0.80 ± 0.33
*Fcgr3a* (CD16A)	5141 ± 2689	3.87 ± 1.08^↑↑↑^	4.12 ± 0.56^↑↑↑^
*Havcr2* (TIM-3)	185 ± 23	28.11 ± 12.41^↑↑↑***^	3.29 ± 0.73^↑↑↑^
*Hvcn1* (Hv1)	1469 ± 183	1.27 ± 0.31	2.81 ± 0.35^↑↑↑***^
*Itgb2*	5351 ± 456	3.17 ± 0.46^↑↑↑***^	0.62 ± 0.08^↓↓↓^
*Msr1* (SR-A, CD204)	4287 ± 844	5.94 ± 0.91^↑↑↑***^	0.12 ± 0.03^↓↓↓^
*Ncf1* (p47phox)	5592 ± 1618	5.43 ± 0.72^↑↑↑^	6.27 ± 1.34^↑↑↑^
*Nox1*	9 ± 3	1.76 ± 0.97	0.53 ± 0.39
*Nox4*	1 ± 0.4	15.84 ± 6.00^↑↑↑***^	3.14 ± 2.02^↑^
*P2rx7*	140 ± 67	0.32 ± 0.19^↓↓**^	1.17 ± 0.55
*P2ry2*	43 ± 8	15.58 ± 2.35^↑↑↑***^	4.62 ± 1.24^↑↑↑^
*P2ry6*	640 ± 270	5.43 ± 0.65^↑↑↑***^	0.70 ± 0.16
*P2ry12*	265 ± 39	0.19 ± 0.16^↓↓↓**^	0.40 ± 0.09^↓^
*Sirpa*	4425 ± 678	0.81 ± 0.17	0.51 ± 0.03^↓↓↓**^


*Treatments, data presentation and analysis are as in **Table 1**.*

##### Unstimulated

Untreated rat microglia are well poised for phagocytosis and ROS production, having moderate to high transcript expression of *Axl*, *Cybb*, *Fcgr1a*, *Fcgr2b*, *Fcgr3a*, *Hvcn1*, *Itgb2, Msr1*, *Ncf1*, and *P2ry6*. In contrast, levels of receptors related to chemotactic migration were low (*P2rx7*, *P2ry2*, *P2ry12*).

##### Stimulated

LPS and I+T both up-regulated *Adora1*, *Adora2a*, *Fcgr3a*, *Havcr2*, *Ncf1*, *Nox4*, and *P2ry2*; however, LPS induced higher levels of *Adora1*, *Havcr2*, *Nox4*, and *P2ry2*. Both stimuli decreased *Axl* and *P2ry12*. Stimulus differences were seen for *Fcgr1a*, *Itgb2*, and *Msr1* (increased by LPS, decreased by I+T); *Fcgr2b* and *P2ry6* (increased by LPS only); *Cybb* and *Hvcn1* (increased by I+T only); *P2rx7* (decreased by LPS only); and *Sirpa* (decreased by I+T only). The 6 h pilot study showed early increases in *Ncf1* and *P2ry2* after either stimulus, while the LPS-mediated induction of *P2ry6* had not yet occurred (Supplementary Table 3).

#### K^+^ and Ca^2+^Channels and Regulators; and Ca^2+^-Signaling Molecules

We examined genes in this category because rodent microglia express numerous potassium (K^+^)- and calcium (Ca^2+^)-permeable channels (and their regulators) and other Ca^2+^-signaling molecules, and Ca^2+^ signaling is crucial for many microglia functions. Several channels, regulators and receptors have been implicated in these responses. For instance, Ca^2+^ entry in rodent microglia is controlled by Ca^2+^-release activated Ca^2+^ (CRAC) channels (comprised of Orai and Stim subunits) under a wide range of conditions (Ohana et al., 2009; Siddiqui et al., 2012; Ferreira and Schlichter, 2013; Lam and Schlichter, 2015; reviewed in Stebbing et al., 2015). Ca^2+^ entry regulates microglial proliferation, migration, phagocytosis and cytokine secretion (Siddiqui et al., 2012; Ferreira and Schlichter, 2013; Heo et al., 2015; Michaelis et al., 2015; reviewed in Parekh, 2010). In addition, Ca^2+^ entry via the reversed mode of the N^+^/Ca^2+^ exchanger regulates ROS production after phagocytosis by rat microglia (Newell et al., 2007). Several Transient Receptor Potential (TRP) channels are expressed in rodent microglia, including TRPM2 (Jeong et al., 2017), TRPM4 (Kurland et al., 2016), and TRPM7 (Jiang et al., 2003; Ohana et al., 2009; Siddiqui et al., 2014). K^+^ channels are increasingly implicated in microglial functions, in part by regulating Ca^2+^ entry, and are considered potential targets for controlling neuroinflammation. Kir2.1 regulates Ca^2+^ signaling, proliferation, and migration (Lam and Schlichter, 2015); Kv1.3 and Kv1.5 regulate proliferation (Kotecha and Schlichter, 1999; Pannasch et al., 2006); Kv1.3 and KCa3.1 regulate ROS production (Khanna et al., 2001; Fordyce et al., 2005; Liu et al., 2013); KCa2.3 and KCa3.1 regulate migration and invasion (D’Alessandro et al., 2013; Ferreira and Schlichter, 2013; Ferreira et al., 2014; Siddiqui et al., 2014; Lam et al., 2017); and Kv1.3, KCa2.3, and KCa3.1 regulate neurotoxicity (Fordyce et al., 2005; Kaushal et al., 2007; Schlichter et al., 2010).

##### Unstimulated

Baseline levels of Ca^2+^-signaling molecules and their regulators were variable. Some were moderately expressed (*Orai1*, *Stim2*, *Trpm2*) while others were lower (*Orai3*, *Stim1*, *Trpm4*, *Trpm7*), and the N^+^/Ca^2+^ exchanger, *Slc8a1*, was expressed at a high level (**Table 5**). Among the K^+^ channels, *Kcnj2* (Kir2.1) was moderately expressed while others were at low levels (*Kcna2*/Kv1.2, *Kcna3*/Kv1.3, *Kcna5*/Kv1.5, *Kcnma1*/KCa1.1/BK/maxi K, *Kcnn3*/KCa2.3, *Kcnn4*/KCa3.1). There was generally moderate expression of most K^+^ channel regulators, including: *Mtmr6* (Myotubularin-related protein 6), *Nme2* (nucleoside diphosphate kinase 2/NDPK2), *Phtp1* (phosphohistidine phosphatase 1/PHP), *Ptpn6* (SHP-1), and *Rest* (RE1 silencing transcription factor). However, there was very high expression of *Calm1* (calmodulin), which regulates the K^+^ channels *Kcnma1*, *Kcnn3*, and *Kcnn4*. It is important to note that despite low mRNA levels, substantial Kv1.3, Kir2.1, and KCa3.1 currents can nonetheless be detected in rat microglia (Kotecha and Schlichter, 1999; Ferreira and Schlichter, 2013; Ferreira et al., 2014; Lam and Schlichter, 2015; Lam et al., 2017; Lively et al., 2018).

**Table 5:** Transcript expression of ion channels and their regulators.

	mRNA counts	Fold change with respect to Control	
		
Gene	Control	LPS	I+T
*Calm1* (CaM)	13457 ± 2110	5.96 ± 1.40^↑↑↑***^	1.75 ± 0.19^↑↑↑^
*Kcna2* (Kv1.2)	42 ± 30	0.63 ± 0.55	0.12 ± 0.07^↓↓∗^
*Kcna3* (Kv1.3)	71 ± 11	1.97 ± 0.69^↑↑^	2.30 ± 0.47^↑↑↑^
*Kcna5* (Kv1.5)	3 ± 1	6.40 ± 6.62	1.73 ± 1.10
*Kcnj2* (Kir2.1)	1239 ± 386	9.04 ± 1.45^↑↑↑**^	5.13 ± 0.92^↑↑↑^
*Kcnma1* (BK)	4 ± 2	3.24 ± 1.77*	0.84 ± 0.70
*Kcnn3* (KCa2.3)	14 ± 10	11.39 ± 6.35^↑↑↑**^	1.47 ± 0.48
*Kcnn4* (KCa3.1)	31 ± 8	1.05 ± 0.96	1.99 ± 0.45
*Mtmr6*	541 ± 85	3.57 ± 0.62^↑↑↑***^	1.56 ± 0.16^↑↑↑^
*Nme2* (NDPK-B)	3166 ± 283	2.90 ± 0.25^↑↑↑***^	0.87 ± 0.11
*Orai1*	658 ± 104	4.56 ± 0.76^↑↑↑***^	1.06 ± 0.11
*Orai3*	280 ± 16	3.59 ± 0.79^↑↑↑***^	2.22 ± 0.11^↑↑↑^
*Phtp1*	581 ± 74	2.28 ± 0.41^↑↑↑***^	1.05 ± 0.11
*Ptpn6* (SHP-1)	1626 ± 257	1.77 ± 0.27^↑↑↑**^	1.20 ± 0.13
*Rest*	589 ± 48	4.40 ± 0.83^↑↑↑^	3.93 ± 0.37^↑↑↑^
*Slc8a1* (NCX1)	3699 ± 516	0.42 ± 0.14^↓↓↓∗∗^	0.68 ± 0.06^↓^
*Stim1*	256 ± 23	2.04 ± 0.56^↑↑↑^	2.23 ± 0.25^↑↑↑^
*Stim2*	547 ± 59	1.57 ± 0.43^↑↑^	1.25 ± 0.17
*Trpm2*	932 ± 541	1.14 ± 0.28	1.73 ± 0.42
*Trpm4*	25 ± 3	3.43 ± 1.31^↑↑↑***^	1.15 ± 0.26
*Trpm7*	439 ± 26	2.54 ± 0.36^↑↑↑***^	1.18 ± 0.06^↑^


*Treatments, data presentation and analysis are as in **Table 1**.*

##### Stimulated

LPS and I+T both increased several Ca^2+^-related molecules (*Orai3*, *Stim1*, *Trpm7*), and K^+^ channels and their regulators (*Calm1*, *Kcna3*, *Kcnj2*, *Mtmr6*, *Rest*). However, LPS increased expression of more genes in this group (15/21, compared with 8/21 for I+T) and the increases were usually higher. Neither stimulus affected *Kcna5*, *Kcnma1*, *Kcnn4*, and *Trpm2* but both decreased *Slc8a1*. Some stimulus differences were that LPS (but not I+T) increased *Kcnn3*, *Nme2*, *Orai1*, *Phtp1*, *Ptpn6*, *Stim2*, and *Trpm4*; and I+T (but not LPS) decreased *Kcna2*. As early as 6 h, similar effects of LPS and I+T were seen for *Kcna3*, *Kcna5*, *Kcnj2*, *Kcnn3*, and *Kcnn4* (Supplementary Table 3).

### Comparing Effects of Subsequent IL-4 or IL-10 Addition After LPS and I+T

**FIGURE 3:**
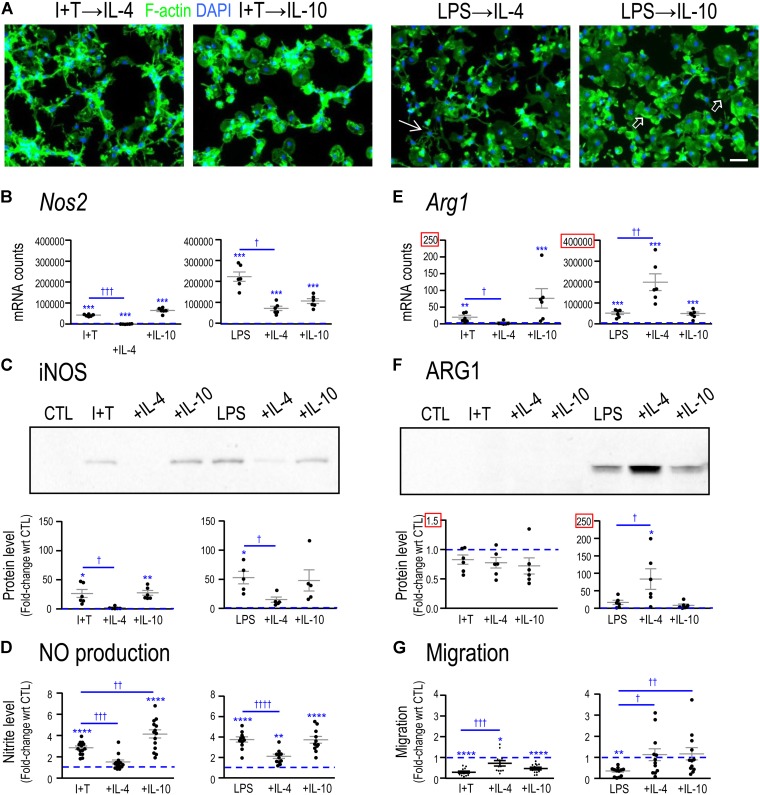
Comparing functional reprogramming by IL-4 or IL-10 after treating with pro-inflammatory stimuli. Primary rat microglia were treated for 2 h with LPS or IFNγ+TNFα followed by a 22 h exposure to IL-4 or IL-10. All data were then obtained at 24 h after the initial treatment. Graphical results are shown for microglia cultures from individual rats, expressed as mean ± SEM. In all graphs, dashed lines indicate mean levels of unstimulated (CTL) microglia. **(A)** Representative confocal micrographs showing microglial morphology. Fixed cells were stained for F-actin (green) and the nuclear marker, DAPI (blue). Examples of unipolar cells (open arrows) and a star-shaped cell (thin arrow) are shown. Scale bar, 50 μm. **(B)** *Nos2* mRNA levels were measured by Nanostring and expressed as mRNA counts/200 ng RNA sample (5–6 individual cultures). **(C)** Western blots show iNOS protein expression (5–6 individual cultures). **(D)** Cumulative nitric oxide (NO) production measured using the Griess assay to quantify levels of nitrite (in μM) (11–14 individual cultures). **(E)** *Arg1* mRNA levels were measured by Nanostring (5–6 individual cultures). **(F)** Western blots show ARG1 protein levels (six individual cultures). **(G)** Microglial migration was measured as cell transit through the 8-μm holes and normalized to untreated (CTL) microglia (dashed line at 1.0) (12 individual cultures). Differences are indicated with respect to control microglia (^∗^) and between stimuli (†). One symbol indicates *p* < 0.05, two symbols, *p* < 0.01, three symbols, *p* < 0.001; four symbols, *p* < 0.0001.

Primary rat microglia were stimulated with LPS or I+T, followed 2 h later by either IL-4 or IL-10, and then functional changes and transcription profiles were examined at 24 h. When microglia were stimulated with LPS followed by IL-4 (LPS→IL-4), there was substantial variability in their morphology (**Figure 3A**). Although there were some round and flat cells that are characteristic of LPS treatment, there were also unipolar cells with a lamellum and trailing process, as well as small-bodied cells with multiple thin branching processes. When IL-10 was added after LPS (LPS→IL-10), cell morphology was also variable but more unipolar cells were observed instead of cells with multiple branching processes. When IL-4 was added after I+T (I+T→IL-4), the morphology remained similar to I+T treatment alone, and cells were mainly unipolar or had multiple short processes. Adding IL-10 to I+T treated cells (I+T→IL-10) changed their morphology to round or round and flat. Rarely did we observe unipolar cells or cells containing multiple processes. In every condition in which I+T was present, chain-like cell groupings were observed.

Functional and molecular responses were affected by IL-4 and, to a lesser degree, by IL-10, and their resolving capacities often depended on the pro-inflammatory stimulus used. In both I+T-treated and LPS-treated microglia, IL-4 (but not IL-10) decreased induction of *Nos2* mRNA (**Figure 3B**), iNOS protein (**Figure 3C**) and NO production (**Figure 3D**). In contrast, for the iNOS-competing enzyme, ARG1, the action of IL-4 depended on the activating stimulus. For LPS-treated (but not I+T treated) cells, IL-4 increased *Arg1* mRNA (**Figure 3E**) and ARG1 protein (**Figure 3F**). Both LPS and I+T reduced migration but the extent of recovery varied (**Figure 3G**). IL-4 partially restored migration of both LPS- and I+T-treated cells, but IL-10 only improved migration of LPS-treated cells. These results suggest significant reprogramming of microglial functions (especially by IL-4) and the next step was to use transcription profiling to assess whether differences in responses to the resolving cytokines after LPS versus I+T stimulation are broad-based or limited to specific classes of outcome.

#### IL-4 Reduced Pro-inflammatory Responses; IL-10 Was Less Effective

**FIGURE 4:**
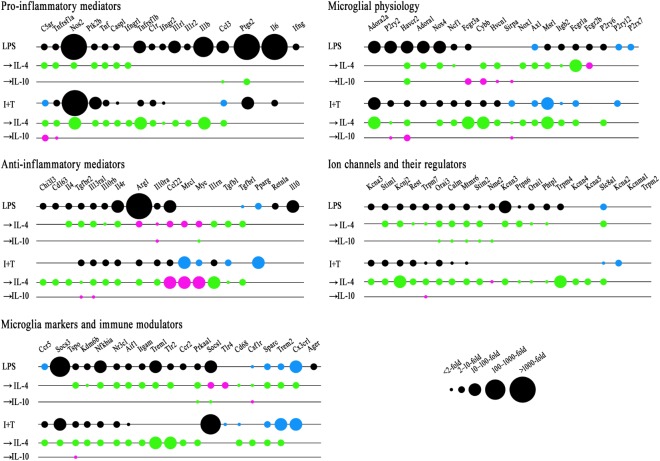
Repolarization by IL-4 or IL-10 of LPS- and IFNγ+TNFα-induced transcript changes. Treatments and NanoString analysis as in **Figure 3**. Transcript levels were expressed as fold changes and analyzed with a two-way ANOVA with Fisher’s LSD test (*p* < 0.05 after FDR correction; *n* = 6–7 individual cultures). See Supplementary Tables 4–8 for full data and significance levels. To create the bubble chart, fold changes were sorted into bins according to the ranges stated on the figure. The chart shows: (1) fold changes evoked by LPS or I+T relative to control values (black = increases; blue = decreases); (2) fold changes caused by IL-4 or IL-10 compared with LPS- or I+T-induced expression (magenta = increases; green = decreases).

**FIGURE 5:**
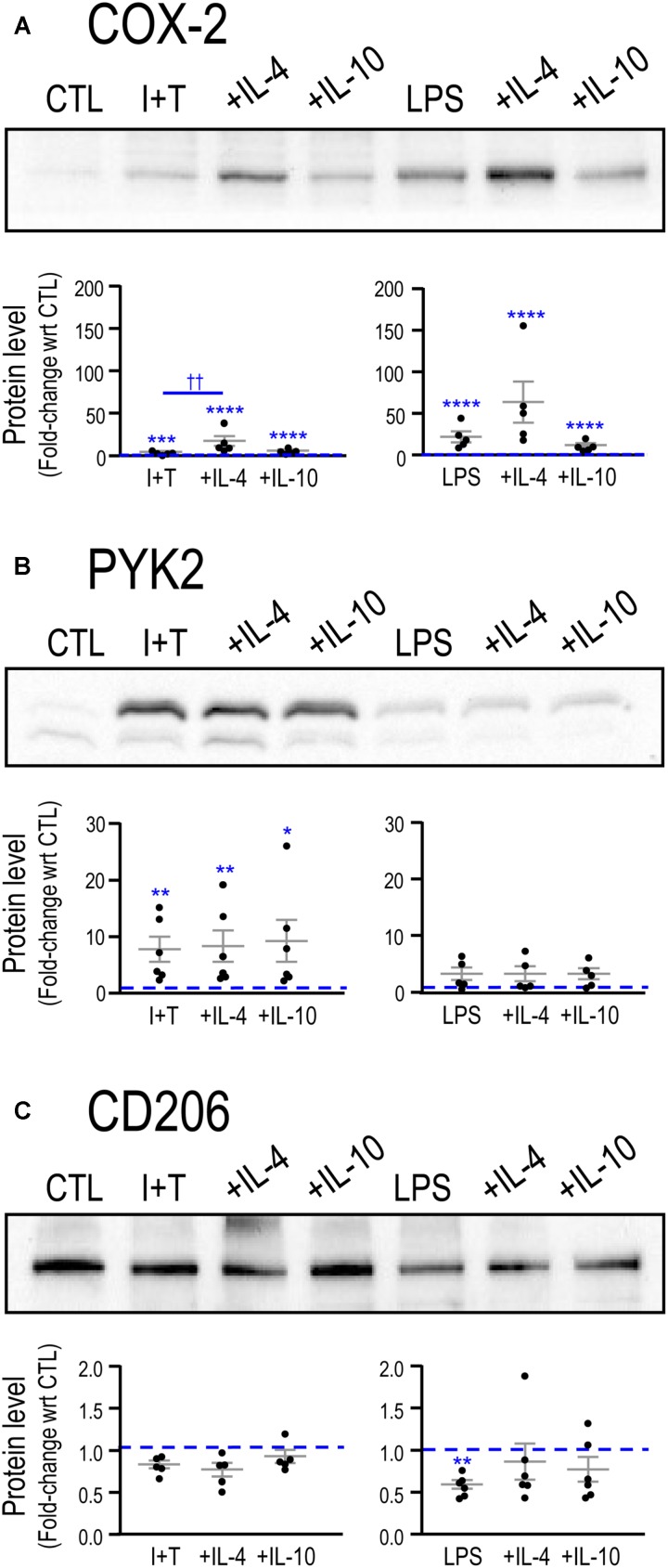
Repolarization of exemplary pro- and anti-inflammatory proteins by IL-4 or IL-10. Representative Western blots and summarized fold-changes in protein with respect to control (CTL; dashed line) for the pro-inflammatory markers, COX-2 **(A)** and PYK2 **(B)**, and the anti-inflammatory marker, CD206 **(C)**. Primary rat microglia were stimulated with LPS or IFNγ+TNFα (I+T) for 2 h before adding IL-4 or IL-10 for a further 22 h. Data are expressed as mean ± SEM (5–6 individual cultures). Differences are indicated with respect to control microglia (^∗^) and between stimuli (†). One symbol of either type indicates *p* < 0.05; two symbols, *p* < 0.01; three symbols, *p* < 0.001; four symbols, *p* < 0.0001.

Adding IL-4 or IL-10 after a pro-inflammatory stimulus reduced expression of some pro-inflammatory molecules but IL-4 was much more effective (**Figure 4**). For instance, 10 pro-inflammatory genes were increased by both LPS and I+T; and IL-4 reduced five of them: *Casp1*, *Nos2*, *Ptk2b*, *Tnf*, *Tnfrsf1a*. In fact, *Casp1* and *Tnfrsf1a* returned to control levels or lower (Supplementary Table 4). Overall, I+T-treated cells showed considerably more plasticity than LPS-treated cells. IL-4 also reduced I+T-mediated increases in *C1r*, *Ifngr2*, and *Tnfrsf1b*. IL-10 evoked little resolution, except for reduced *Ccl3* and *Ptgs2* in LPS-treated cells, and instead, it amplified the I+T-mediated increase in *Tnfrsf1a*. Other examples of lower plasticity of LPS-treated cells were that neither IL-4 nor IL-10 affected the increases in *Ifngr2*, *Il1b*, *Il1r1*, *Il1r2*, *Il6*, and *Tnfrsf1b*. Resolution of pro-inflammatory responses seen at the transcript level at 24 h was not necessarily accompanied by protein changes at that time. For example, in both LPS- and I+T-treated cells, IL-4 increased COX-2 (**Figure 5A**) and failed to decrease PYK2 (**Figure 5B**). In future, it would be interesting to compare the time-course of changes in transcript and protein levels in case resolution at the protein level simply takes longer.

#### IL-4 and IL-10 Effects on Anti-inflammatory Genes and Receptors

In **Table 2**, we showed that LPS, and to a lesser degree, I+T, increased several molecules associated with an anti-inflammatory phenotype. Here, we found that IL-4 altered anti-inflammatory gene expression after LPS or I+T treatment, but some genes increased and some decreased (**Figure 4** and Supplementary Table 5). For both LPS- and I+T-treated microglia, IL-4 increased expression of the alternative-activation markers, *Ccl22*, *Myc*, and *Mrc1* (but without affecting CD206 protein at 24 h; **Figure 5C**). Surprisingly, IL-4 decreased several genes and receptors for resolving cytokines in both conditions (*Il1rn*, *Il4*, *Il4r*, *Il10rb*, *Il13ra1*, *Tgfb1*, *Tgfbr1*, *Tgfbr2*). Some differences depended on the pro-inflammatory stimulus used. In LPS-treated cells, IL-4 increased *IL-10ra* and *Arg1*; however, in I+T-treated cells, IL-4 reduced *IL-10ra* and restored *Arg1* to control levels. In I+T-treated cells only, IL-4 decreased *Cd163* and *Chi3l3*. IL-10 was less effective in altering microglial responses but it selectively reduced *Myc* in LPS-treated cells and increased *Il13ra1* and *Tgfbr2* in I+T-treated cells. Genes that did not show alterations by IL-4 or IL-10 include *Pparg* (decreased by LPS and I+T) and *Cd163*, *Il10* and *Retnla* (increased by LPS only).

#### IL-4 and IL-10 Effects on Microglial Activation Markers and Immune Modulators

IL-4 substantially reduced most genes that were increased by the pro-inflammatory stimuli. Of the seven genes that were increased by LPS and I+T, IL-4 reduced five of them (*Aif1*, *Kdm6b*, *Nfkbia*, *Nr3c1*, *Tspo*) (**Figure 4** and Supplementary Table 6). IL-4 reduced the I+T-specific induction of *Ccr5* to below control levels. Among the genes selectively increased by LPS, IL-4 reduced *Itgam*, *Prkaa1*, *Trem1*, and *Tlr2*. Although these genes were not elevated after I+T, IL-4 reduced their expression to below control levels. IL-4 decreased several other genes that had not been affected or were already reduced: *Cd68*, *Csf1r*, *Sparc*, *Trem2* (both conditions), and *Cx3cr1*(LPS only). In LPS-treated cells, IL-4 increased *Socs1* and *Tlr4*; whereas, after I+T, no genes were increased by IL-4. IL-10 had fewer effects and some were opposite to IL-4. IL-10 decreased *Socs1* and *Prkaa1* and increased *Csf1r* after LPS treatment; and after I+T, it increased *Tspo*. Genes that did not show alterations include *Ager*, *Ccr2*, *Ccr5*, and *Socs3* after LPS; *Cx3cr1*, *Socs1*, and *Tlr4* after I+T.

#### IL-4 and IL-10 Effects on Genes Related to Microglia Physiological Functions

Again, IL-4 affected more genes than did IL-10, and often reduced both control and up-regulated levels (**Figure 4** and Supplementary Table 7). For both LPS- and I+T-treated cells, IL-4 reduced 10/19 genes in this category: *Axl*, *Cybb*, *Fcgr1a*, *Hvacr2*, *Hvcn1*, *Itgb2*, *Msr1*, *Ncf1*, *Nox1*, and *Nox4*. The down-regulated genes include receptors involved in phagocytosis and mediators of ROS production. Again, there were more counteracting effects in I+T-treated cells than after LPS; e.g., decreased *Adora2a*, *Fcgr3a*, and *P2ry2* expression after I+T only. Reduced *Adora1* was specific to LPS-treated cells. IL-4 increased expression of *Fcgr2b* in LPS-treated cells and *P2ry6* in I+T-treated cells. As above, actions of IL-10 were often opposite to IL-4; e.g., IL-10 increased *Sirpa* (both conditions); *Cybb*, *Fcgr3a*, *Hvcn1* (after LPS), *Havcr2*, and *Pr2y2* (after I+T). *P2rx7* and *P2ry12* were unaffected by IL-4 and IL-10 in either condition.

#### IL-4 and IL-10 Effects on K^+^ and Ca^2+^ Channels and Regulators; and Ca^2+^-Signaling Molecules

Again, IL-4 affected more genes than IL-10 (**Figure 4**). In both LPS- and I+T-treated microglia, IL-4 reduced expression of 13/21 genes: *Calm1*, *Kcnj2*, *Kcnn3*, *Mtmr6*, *Orai1*, *Orai3*, *Phtp1*, *Ptpn6*, *Rest*, *Slc8a1*, *Stim1*, *Stim2*, and *Trpm7*. Some gene levels were then below control values: *Stim 1* and *Stim2* (both conditions); *Kcnj2*, *Kcnn3*, *Mtmr6*, *Orai3*, *Rest*, *Trpm7* (after I+T), and *Ptpn6* (after LPS) (Supplementary Table 8). IL-4 decreased *Slc8a1* expression further beyond the decrease mediated by LPS or I+T. IL-4 also decreased *Kcna3*, *Kcna5*, *Kcnn4*, and *Trpm4* (after I+T). By decreasing genes that regulate both K^+^ and Ca^2+^ signaling, this finding raises the possibility that secondary exposure to IL-4 dampens the contribution of these ion channels to subsequent microglial stimuli. The only increase mediated by IL-4 was elevated *Nme2* after I+T. IL-10 again had few effects, and only in LPS-treated cells; i.e., decreased *Calm1*, *Mtmr6*, *Nme2*, *Orai3*, and *Stim2* (LPS only) and increased *Trpm7* (I+T only).

## Discussion

LPS, which is a cell wall component of *E. coli* bacteria, binds to TLR4 and activates NFκB signaling (Hoshino et al., 1999). It is also possible that endogenous TLR4 ligands are released after damage and elicit inflammatory responses (reviewed in Yu et al., 2010). While responses to LPS are often used to describe microglial pro-inflammatory reactions, rarely are other stimuli compared. As also explained in the Introduction, we chose to compare LPS with IFNγ+TNFα for several reasons. In the healthy CNS, the normally low TNFα levels regulate synaptic function and plasticity (Stellwagen and Malenka, 2006; reviewed in Santello and Volterra, 2012). When TNFα is increased after CNS damage, it can initiate harmful pro-inflammatory microglial responses involving NFκB and Activator Protein 1 (AP-1) signaling (Kroner et al., 2014; reviewed in MacEwan, 2002). IFNγ also increases in CNS damage and disease states, and while it was originally thought to derive from circulating lymphocytes, microglia and astrocytes also produce IFNγ (Xiao and Link, 1998; Suzuki et al., 2005; Kawanokuchi et al., 2006). IFNγ can alter microglial reactivity and potentiate glial responses to other cytokines (Jensen et al., 2000; Blais and Rivest, 2004; Mir et al., 2008; Mir et al., 2009). The second aspect of this study addressed the potential plasticity of microglial reactive states, an area where not enough is known. Discussion of our results and the literature will be divided into two sections: microglial molecular and functional responses to LPS and I+T; and the resolving capacity of the anti-inflammatory cytokines, IL-4 and IL-10.

### Comparing Effects of LPS and I+T

LPS has been well studied (reviewed in Perry and Andersson, 1992; Lund et al., 2006; Wang et al., 2006; Hoogland et al., 2015) and is a particularly potent activator of inflammatory responses. However, there is increasing evidence that it evokes a mixed gene profile, rather than strictly pro-inflammatory. For instance, LPS up-regulated IL-10 signaling molecules in primary mouse microglia (Das et al., 2017), increased IL-10 secretion from rat microglia (Ledeboer et al., 2002), and increased the hallmark “alternative activation” markers, *Ccl22* and *Arg1*, in primary mouse and rat microglia, respectively (Columba-Cabezas et al., 2002; Zhang et al., 2009). *In vivo*, there was concurrent elevation of IL-1β and IL-10 transcripts in the mouse cerebral cortex at 8 h after intraperitoneal LPS injection (Henry et al., 2009). Overall, LPS appears to evoke a much broader inflammatory profile than previously thought. One possibility is that induction of anti-inflammatory cytokines results in local autocrine/paracrine regulation of pro-inflammatory actions.

A functional characteristic of pro-inflammatory microglial states appears to be reduced migration, as seen for both LPS and I+T treatment (Lively and Schlichter, 2013; Lam et al., 2017; present study). Here, both stimuli increased numerous pro-inflammatory genes, which is not surprising given that LPS and I+T activate NFκB; however, some LPS-evoked changes were greater and some differed from I+T. Such differences in their reactive phenotype raises the possibility that their susceptibility to subsequent stimuli might also differ. A few examples follow. (1) LPS up-regulated *Il1b* and its receptor, *Il1r1*, which promote IL-1 responses; whereas, I+T elevated the IL-1 antagonist, *Il1rn*, which might render these cells less responsive. (2) LPS decreased expression of *Ccr5*, a proposed decoy receptor for pro-inflammatory chemokines (Doodes et al., 2009), while the increase with I+T might make these cells less responsive to CCR5 ligands (CCL3, CCL4, CCL5). (3) There was much greater iNOS induction by LPS than I+T but nitric oxide production was only slightly higher. However, LPS also increased ARG1 mRNA and protein and, because ARG1 competes with iNOS for the substrate arginine (Rath et al., 2014), this might represent a stimulus-specific regulatory mechanism to limit NO production. (4) LPS also increased other molecules classified as anti-inflammatory, including *Ccl22*, *Il4* and *Il10*.

Down-regulated genes are rarely discussed when describing microglial reactions to pro-inflammatory stimuli. However, this area of investigation has been facilitated by recent high-content molecular studies that have identified homeostatic signature genes (Gautier et al., 2012; Chiu et al., 2013; Orre et al., 2014; Zhang et al., 2014), surface receptors and molecules that constitute a “sensome” (Hickman et al., 2013), and disease-associated profiles (Keren-Shaul et al., 2017; Hirbec et al., 2018). Down-regulation of homeostatic molecules in microglia appears to indicate a switch from surveillance activities to reactive responses. We found that both LPS and I+T decreased several homeostatic signature molecules: *Trem2*, *Cx3cr1*, *P2ry12*, and *Sparc*. This is consistent with several *in vivo* studies. For instance, the matricellular molecule, SPARC, was down-regulated in reactive mouse microglia after photo-thrombotic cortical ischemia and excitotoxic olfactory bulb lesions (Lloyd-Burton et al., 2013). P2RY12, which is an important sensor of extracellular nucleotides released early after damage, was reduced in activated microglia after LPS injection into the striatum (Haynes et al., 2006) or peritoneal cavity (Hirbec et al., 2018), and in microglia within human MS lesions (Zrzavy et al., 2017). CX3CR1, which helps maintain microglia in a non-reactive state *in vivo* (Bachstetter et al., 2011), decreased after intraperitoneal LPS injection in mice (Wynne et al., 2010; Hirbec et al., 2018). There was also reduced microglial expression of CX3CR1 and P2RY12 as they adopted a disease-associated phenotype in mouse models of Alzheimer’s disease and MS (Keren-Shaul et al., 2017). Based on these results, and the profound decreases in P2RY12 and CX3CR1 we observed after LPS or I+T treatment, their use in distinguishing resident microglia from peripheral macrophages (Haynes et al., 2006; Butovsky et al., 2014) might not be reliable under pathological conditions.

TREM2, CX3CR1, and P2RY12 are also members of the sensome, a large group of cell surface receptors that microglia use to detect changes in their environment (Hickman et al., 2013). Changes in sensome-related molecules with microglial activation or under disease states are only beginning to be characterized. Decreases in their expression under pro-inflammatory conditions could alter the ability of microglia to detect subsequent environmental changes. Of the 95 genes we examined, 40 are cell surface receptors that detect nucleotides, cytokines, chemokines, ECM molecules, and cellular debris; and are consistent with the sensome classification. We found that LPS and I+T changed expression of many of these molecules, including increases in multiple purinergic and phagocytosis receptors. Similar changes in sensome gene expression were seen in mouse microglia isolated 24 h after intraperitoneal LPS injection (Hirbec et al., 2018); e.g., increases in *C5ar1*, *Fcgr1*, *Fcgr2b*, *Fcgr3a*, *Il4ra*, *Itgb2*, *P2ry6*, *Tlr2*, and *Tnfrsf1a*; and decreases in *Csf1r*, *Cx3cr1*, *P2ry12*, and *Tgfbr1*. In addition, both pro-inflammatory stimuli, but especially LPS, increased several ion channels (cell-surface molecules that affect microglial functions) and their regulators and, perhaps in future, ion channels should be considered as members of the sensome. It is reasonable to hypothesize that expression of sensome-related genes will change depending on injury severity, as well as the disease type and stage. However, much more information is needed from *in vivo* disease and damage models.

### Resolving Capacity After Pro-inflammatory Stimuli

In most forms of CNS damage and disease, monocytes/macrophages infiltrate, and these cell types are known for their malleable responses that depend on the stimuli they encounter (Stout et al., 2005; Gratchev et al., 2006). The extracellular milieu is particularly dynamic after acute CNS damage and microglia can be exposed to cell debris, intracellular contents, ionic disturbances, and numerous inflammatory mediators. With so many potential stimuli, it is difficult to determine from *in vivo* damage models how microglia respond to individual or changing stimuli. Thus, it is useful to assess changes in receptors for damage- and disease-related mediators. While LPS and I+T appear to broadly prime microglia for a subsequent stimulus exposure, there were also stimulus-specific changes in receptor expression. For instance, we found that LPS (but not I+T) elevated *Ager* and *Trem1*, receptors that perpetuate pro-inflammatory profiles (reviewed in Wilkinson and El Khoury, 2012; Tammaro et al., 2017). In contrast, I+T reduced the LPS receptor, *Tlr4*, and evoked earlier increases in the receptors for IL-4, *Il4r*, and *Il13ra1*. Hence, we asked whether I+T-treated microglia are more responsive to the resolving cytokines, IL-4 and IL-10.

Our IL-4 results are consistent with a skewing toward refractory or alternative activation states. IL-4 better resolved responses to I+T than LPS, often decreased expression of pro-inflammatory receptors to below control levels, and increased expression of hallmark alternative activation markers (*Ccl22*, *Mrc1*, *Myc*) in both LPS- and I+T-treated microglia. Most previous reports used a limited panel of sentinel molecules and found that IL-4 reduced LPS-evoked pro-inflammatory mediators and/or increased alternative activation markers (Kitamura et al., 2000; Cao et al., 2007; Chhor et al., 2013; Peferoen et al., 2015). However, in one study, IL-4 did not reduce LPS-mediated secretion of TNFα or IL-1β (Wirjatijasa et al., 2002). We now extend the information to I+T stimulation, and also show changes in expression of many genes, including molecules related to microglial physiological functions, Ca^2+^ signaling and ion channels.

IL-10 is usually considered a resolving cytokine for pro-inflammatory microglial states. We found that IL-10 was not as effective as IL-4 and that its resolving actions depended on the initial stimulus. IL-10 restored levels of fewer genes, was less effective in restoring migration, and was especially ineffective after I+T treatment, failing to reduce expression of any of the up-regulated genes examined. Surprisingly, IL-10 increased NO production. Other comparisons with the literature are limited to resolution of LPS-mediated responses. Differences between studies might depend on the order of cytokine exposure, time examined, outcome measures used, presence of other cell types, and rodent species. For primary mouse microglia, IL-10 pre-treatment was more effective than IL-4 in reducing subsequent LPS-mediated secretion of IL-1α, IL-1β, IL-6, and CCL2 (Szczepanik et al., 2001). That study did not examine transcript or protein levels. Pre-treatment with IL-10 reduced LPS-mediated increases in production of TNFα and reactive nitrogen and oxygen species from rat microglia (Qian et al., 2006). The presence of astrocytes might affect IL-10-mediated resolution, as they appear to be more responsive than microglia to IL-10 *in vitro*. LPS reduced expression of numerous inflammatory mediators in astrocytes; and it increased Transforming growth factor β1 (TGFβ1), which in turn, dampened microglial responses to LPS (Norden et al., 2014). In LPS-treated rat astrocyte-microglia co-cultures, IL-10 then reduced production of NO, IL-1β, IL-6, and TNFα (Ledeboer et al., 2000). Rodent age might also be a factor in IL-10 responsiveness. Astrocytes from aged mice have lower expression of the IL-10 receptor and TGFβ1; and these cytokines are less effective in resolving microglial inflammatory responses after systemic LPS injection (Norden et al., 2016). Thus, IL-10 might preferentially dampen LPS-mediated pro-inflammatory actions in an astrocyte-dependent manner. We used essentially pure microglial cultures, and this might contribute to the smaller IL-10 effects we observed. Nevertheless, because IL-10 altered microglial morphology and restored their migration in LPS-treated cells, some effects on microglia are cell autonomous.

## Conclusion and Future Directions

(i) LPS, which is the most common stimulus used to investigate microglial reactivity and pro-inflammatory responses, actually produces a mixed inflammatory outcome. This mixed profile was less pronounced when using IFNγ+TNFα, and this stimulus was more simply pro-inflammatory. There is evidence that microglia display mixed profiles *in vivo*, with increases in both pro- and anti-inflammatory molecules. This has been seen in rodent models of traumatic brain injury (e.g., Kim et al., 2016; Morganti et al., 2016) and stroke (e.g., Li et al., 2001; Wasserman et al., 2007; Sieber et al., 2011; Lively et al., 2016). While there has already been considerable transcriptional profiling of LPS-treated microglia, different programs of activation and gene expression in response to differing stimuli require further study of endogenous stimuli. (ii) There is concern that microglial activation *in vitro* does not reflect *in vivo* responses. The finding that responses to LPS and other “classical” activators differ and are complicated might help account for discrepancies between *in vitro* and *in vivo* results. (iii) There is increasing information about how the molecular composition of the extracellular space evolves after CNS damage. However, not enough is known about the potential for mixed microglial responses and how mechanisms to regulate their subsequent behavior might depend on the specific stimuli and sequence of exposure. We found that the dampening effects of the two resolving cytokines (IL-4, IL-10) differed, and also depended on the pro-inflammatory stimulus. Future studies should consider effects of other pro-inflammatory stimuli (e.g., IL-1β), and potential competition or resolution by other anti-inflammatory stimuli (e.g., TGFβ1). Studies performed *in vitro* that delineate multi-faceted microglial responses to individual inflammatory mediators can help inform *in vivo* studies, and together increase our understanding of microglial contributions to CNS pathology.

## Data Availability

The raw data supporting the conclusions of this manuscript are found in the Dryad Digital Repository (https://doi.org/10.5061/dryad.st860bt).

## Author Contributions

SL and LS conceived the project and wrote the manuscript. SL carried out the experiments and data analysis.

## Conflict of Interest Statement

The authors declare that the research was conducted in the absence of any commercial or financial relationships that could be construed as a potential conflict of interest.

## Footnotes

1 https://www.pmgenomics.ca/pmgenomics
