# Supplementary material for: Microglia Responses to Pro-inflammatory Stimuli (LPS, IFNγ+TNFα) and Reprogramming by Resolving Cytokines (IL-4, IL-10)
Source: Front Cell Neurosci. 2018 Jul 24;12:215. doi: 10.3389/fncel.2018.00215 (PMC6066613; doi:10.3389/fncel.2018.00215)
Supplement: Supplementary file 1 [file Table_1.pdf]

Microglia responses to pro-inflammatory stimuli (LPS, IFNγ + TNFα) and reprogramming by resolving cytokines (IL-4, IL-10)

Starlee Lively and Lyanne C. Schlichter*

* Correspondence: Professor Lyanne C. Schlichter Lyanne.Schlichter@uhnresearch.ca

Supplementary Table 1. Target sequences of genes used to create Custom CodeSet for nCounter Assay (6 h)

Gene	Accession #	Target sequence
Arg1	NM_017134.2	ACGGGAAGGTAATCATAAGCCAGAGACTGACTACCTTAAACCACCGAAAT<br>AAATGTGAATACATCGCATAAAAGTCATCTGGGGCATCACAGCAAACCGA
Casp1 (ICE)	NM_012762.2	AGATTCTAAGGGAGGACATCCTTTCTCCTCAGAAACAAAAGAAAAACTGA<br>ACAAAGAAGGTGGCGCATTTCCTGGACCGAGTGGTTCCCTCAAGTTTTGC
Ccl22	NM_057203.1	TACATCCGTCACCCTCTGCCACCACGTTTCGTGAAGGAGTTCTACTGGAC<br>CTCAAAGTCCTGCCGCAAGCCTGGCGTCGTTTTGATAACCATCAAGAACC
Cd163	NM_001107887.1	AGTTTCCTCAAGAGGAGAGGTCTTGATACATCAAGTTCAGTACCAAGAGA<br>TGGATTCGAAGACGGATGATCTGGACTTGCTGAAATCCTCGGGTTGGCAT
Cd68	NM_001031638.1	CTCTCATTCCCTTACGGACAGCTTACCTTTGGATTCAAACAGGACCGACA<br>TCAGAGCCACAGTACAGTCTACCTTAACTACATGGCAGTGGAATACAATG
Chi3l3 (Ym1)	NM_001191712.1	AAGCGTTTGAGAAAGAATCTACTGAGCAAGAAATCCCAAGGCTGCTTCTC<br>ACTGCCACAGTAGCTGGAGTCATTGACACAATCCAGTCTGGTTACAAGAT
Cx3cr1	NM_133534.1	ATGTGCAAGCTCACGACTGCTTTCTTCTTCATTGGCTTCTTTGGGGGCAT<br>ATTCTTCATCACCGTCATCAGCATCGACCGGTACCTCGCCATCGTCCTGG
Hprt1	NM_012583.2	AGCTTCCTCCTCAGACCGCTTTTCCCGCGAGCCGACCGGTTCTGTCATGT<br>CGACCCTCAGTCCCAGCGTCGTGATTAGTGATGATGAACCAGGTTATGAC
Il1b	NM_031512.1	TGCACTGCAGGCTTCGAGATGAACAACAAAAATGCCTCGTGCTGTCTGAC<br>CCATGTGAGCTGAAAGCTCTCCACCTCAATGGACAGAACATAAGCCAACA
Il1r2	NM_053953.1	CCACTGTGAACAAATGTCTCTGGAACTCAAGGTCTTTAAGAATACCGAAG<br>CCTCTTTCCCTCTCGTCTCCTACTTGCAAATCTCAGCTCTCTCCTCCACC
Il1rn	NM_022194.2	TCATTGCTGGGTACTTACAAGGACCAAATACCAAACTAGAAGAAAAGATA<br>GACATGGTGCCTATTGACTTTCGGAATGTGTTCTTGGGCATCCACGGGGG
Il4	NM_201270.1	TGCTGTCACCCTGTTCTGCTTTCTCATATGTACCGGGAACGGTATCCACG<br>GATGTAACGACAGCCCTCTGAGAGAGATCATCAACACTTTGAACCAGGTC
Il4r	NM_133380.2	GGGTGTCAGCATCTCCTGCATCTGCATCCTATTGTTTTGCCTGACCTGTT<br>ACTTCAGCATTATCAAGATTAAGAAGATATGGTGGGACCAGATTCCCACT
Il6	NM_012589.1	GGAACAGCTATGAAGTTTCTCTCCGCAAGAGACTTCCAGCCAGTTGCCTT<br>CTTGGGACTGATGTTGTTGACAGCCACTGCCTTCCCTACTTCACAAGTCC
Il10	NM_012854.2	ACAACATACTGCTGACAGATTCCTTACTGCAGGACTTTAAGGGTTACTTG<br>GGTTGCCAAGCCTTGTCAGAAATGATCAAGTTTTACCTGGTAGAAGTGAT
Il13ra1	NM_145789.2	TAACGAATTTGAGTGTCTCTGTCGAAAATCTCTGCACAATAGTGTGGACA<br>TGGAGTCCTCCTGAGGGAGCCAGTCCAAATTGCAGTCTCAGATATTTTAG
Itgam (CD11b)	NM_012711.1	CATCCCTTCCTTCAACAGTAAAGAAATATTCAACGTCACCCTCCAGGGCA<br>ATCTGCTATTTGACTGGTACATCGAGACTTCTCATGACCACCTCCTGCTT
Kcna3 (Kv1.3)	NM_019270.3	GCCACCTTCTCCAGAAATATCATGAACCTGATAGACATTGTAGCCATCAT<br>CCCTTATTTTATTACTCTGGGCACTGAGCTGGCTGAGCGACAGGGTAATG
Kcna5 (Kv1.5)	NM_012972.1	ATCAGAAGGGGTAGCTGTCCTCTAGAAAAGTGTCACCTCAAGGCCAAGAG<br>CAACGTGGACTTGCGGAGGTCCCTGTATGCCCTCTGTCTGGACACTAGCC
Kcnj2 (Kir2.1)	NM_017296.1	GTTCTTTGGCTGTGTGTTTTGGTTGATAGCTCTGCTCCACGGGGATCTGG<br>ATGCTTCTAAAGAGAGCAAAGCGTGTGTGTCTGAGGTCAACAGCTTCACG
Kcnn3 (SK3)	NM_019315.2	AGAGAAAGCGACTGAGTGACTATGCTCTGATTTTTGGGATGTTTGGAATT<br>GTTGTTATGGTGATAGAGACCGAACTGTCTTGGGGTTTGTACTCAAAGGA

Kcnn4<br>(SK4)	NM_023021.1	ATCGGACTCATGGTGCTGCACGCTGAGATGTTGTGGTTCCTGGGTTGCAA<br>GTGGGTGCTGTACCTGCTCTTGGTTAAGTGTTTAATCACGCTGTCCACTG
Mrc1<br>(CD206)	NM_001106123.1	CTTTGGAATCAAGGGCACAGAGCTATATTTTAACTATGGCAACAGGCAAG<br>AAAAGAATATCAAGCTTTACAAAGGTTCCGGTTTGTGGAGCAGATGGAAG
Myc	NM_012603.2	ACCGAGGAAAACGACAAGAGGCGGACACACAACGTCTTGGAACGTCAGAG<br>GAGAAACGAGCTGAAGCGTAGCTTTTTTGCCCTGCGCGACCAGATCCCTG
Ncf1	NM_053734.2	TCCATTCCCAGCATCCCATAATTGGGCTTGTCCGTGTTCCAACATCTGGG<br>CGGAATTTCACAGCCAAAGGTCAAGAGGACTGCTGTTACGTTCAAGGTCG
Nos2<br>(iNOS)	NM_012611.2	ACGGGACACAGTGTCGCTGGTTTGAAACTTCTCAGCCACCTTGGTGAGGG<br>GACTGGACTTTTAGAGACGCTTCTGAGGTTCCTCAGGCTTGGGTCTTGTT
P2rx7	NM_019256.1	ACTTTAAGAGGTCACATTAACCAGACTAGAAGCCATCGCATCTAACCGCA<br>TACCAGACACAGTCTGACGCCTCATTGCTATGCTATGGTTCTAAGTGACT
P2ry2	NM_017255.1	GAGCTCTTTAGCCATTTTGTGGCTTACAGCTCTGTCATGCTGGGTCTGCT<br>TTTTGCTGTGCCCTTTTCCATCATCCTGGTCTGTTACGTGCTCATGGCCC
P2ry6	NM_057124.2	ACGGGATACTCCACCTTGGCTTCACGGTGCCTCCTAATTTTGTAATGCCG<br>TACACACTTCTGTCACGAAAACTTTGTCCACGAGTCCCAAAGTCAAGTTC
Pparg	NM_013124.1	TTTATAGCTGTCATTATTCTCAGTGGAGACCGCCCAGGCTTGCTGAACGT<br>GAAGCCCATCGAGGACATCCAAGACAACCTGCTGCAGGCCCTGGAACTCC
Retnla<br>(FIZZ1)	NM_053333.1	AGGAACTTCTAGCCCATCAAGATAACTATCCCTCTGCTGTAAGGAAGACC<br>CTCTCATGCACTAATGTCAAGTCTATGAGCAAATGGGCCTCCTGCCCTGC
Sdha	NM_130428.1	CCTCCGATTAAGGCAAATGCTGGAGAAGAGTCGGTTATGAATCTTGACAA<br>GTTGAGATTTGCTGATGGAAGTGTAAGAACATCAGAGCTGCGCCTCAGCA
Tgfb1	NM_021578.2	CGCCTGCAGAGATTCAAGTCAACTGTGGAGCAACACGTAGAACTCTACCA<br>GAAATATAGCAACAATTCCTGGCGTTACCTTGGTAACCGGCTGCTGACCC
Tlr2	NM_198769.2	TTTACAAACCCTTAGGGTAGGAAATGTTGACACTTTCAGTGAGATAAGGA<br>GAATAGATTTTGCTGGGCTGACCTCTCTCAACGAACTTGAAATTCAGGTA
Tlr4	NM_019178.1	GTCAGTGTGCTTGTGGTAGCCACTGTAGCATTTCTGATATACCACTTCTA<br>TTTTCACCTGATACTTATTGCTGGCTGTAAAAAGTACAGCAGAGGAGAAA
Tnf	NM_012675.2	GGTGATCGGTCCCAACAAGGAGGAGAAGTTCCCAAATGGGCTCCCTCTCA<br>TCAGTTCCATGGCCCAGACCCTCACACTCAGATCATCTTCTCAAAACTCG
Ywhaz	NM_013011.3	TCCTGAACTCCCCAGAGAAAGCCTGCTCTCTTGCAAAAACAGCTTTTGAT<br>GAAGCCATTGCTGAACTTGATACATTAAGTGAAGAGTCGTACAAAGACAG
