# Supplementary material for: Microglia Responses to Pro-inflammatory Stimuli (LPS, IFNγ+TNFα) and Reprogramming by Resolving Cytokines (IL-4, IL-10)
Source: Front Cell Neurosci. 2018 Jul 24;12:215. doi: 10.3389/fncel.2018.00215 (PMC6066613; doi:10.3389/fncel.2018.00215)
Supplement: Supplementary file 2 [file Table_2.pdf]

Microglia responses to pro-inflammatory stimuli (LPS, IFNγ + TNFα) and reprogramming by resolving cytokines (IL-4, IL-10)

Starlee Lively and Lyanne C. Schlichter*

* Correspondence: Professor Lyanne C. Schlichter Lyanne.Schlichter@uhnresearch.ca

Supplementary Table 2. Rat target sequences used to create CodeSet for nCounter Assay (24 h)

Gene	Accession #	Target sequence
Adora1	NM_017155.2	TGTCCTCATCCTCACCCAGAGCTCCATTCTGGCTCTGCTCGCCATTGCTG TGGATCGATACCTCCGAGTCAAGATCCCTCTCCGGTACAAGACAGTGGTG
Adora2a	NM_053294.3	CCCTGGAGAAGTCATGATTTTGGGAGATGCAGAGTCGTCTGTGAGAACGT CTTCGGGGAGCTCTCTCTGGGAACGCTCGTGGCTCTTGTGAGGAAAGGGC
Ager (Rage)	NM_053336.2	AACTAACAGGCTTGGGAAGGAGGTCAAGTCCAACTACCGAGTCCGAGTCT ACCAGATTCCTGGGAAGCCGGAAATTGTGAATCCTGCCTCTGAACTCACA
Aif1 (Iba1)	NM_017196.2	ATCGATATTATGTCCTTGAAGCGAATGCTGGAGAAACTTGGGGTTCCCAA GACCCATCTAGAGCTGAAGAAATTAATTAGAGAGGTGTCCAGTGGCTCCG
Arg1	NM_017134.2	ACGGGAAGGTAATCATAAGCCAGAGACTGACTACCTTAAACCACCGAAAT AAATGTGAATACATCGCATAAAAGTCATCTGGGGCATCACAGCAAACCGA
Axl	NM_001013147.1	GGAAGAAGGAGACGAGATATGGGGAGGTGTTCGAGCCAACTGTGGAAAGG GGTGAACTGGTAGTCAGGTACCGTGCCCGAAAGTCCTACAGTCGCCGGAC
C1r	XM_001061611.1	ACAAAGACCTTATGGGTTATGTCAGCGGCTTCGGGATAACAGAAGATAAA ATAGCTTTTAATCTCAGGTTTGTCCGTCTGCCCATAGCCGATCGAGAGGC
C5ar1	NM_053619.1	GTACTGTCAACGCCATTTGGTTTCTGAATCTGGCGGTGGCCGACCTCCTC TCGTGCTTGGCACTGCCTATCCTGTTTACGTCCATTGTAAAGCATAACCA
Calm	NM_031969.2	GCTACATCAGTGCGGCAGAACTGCGCCACGTCATGACAAACCTCGGGGAA AAGCTAACAGATGAAGAAGTAGACGAAATGATCAGAGAAGCAGATATTGA
Casp1 (ICE)	NM_012762.2	AGATTCTAAGGGAGGACATCCTTTCTCCTCAGAAACAAAAGAAAAACTGA ACAAAGAAGGTGGCGCATTTCCTGGACCGAGTGGTTCCCTCAAGTTTTGC
Ccl3	NM_013025.2	ACGGCAAATTCCACGAAAATTCATTGCTGACTATTTTGAGACCAGCAGCC TTTGCTCCCAGCCGGGTGTCATTTTCCTGACCAAGAGAAACCGGCAGATC
Ccl22	NM_057203.1	TACATCCGTCACCCTCTGCCACCACGTTTCGTGAAGGAGTTCTACTGGAC CTCAAAGTCCTGCCGCAAGCCTGGCGTCGTTTTGATAACCATCAAGAACC
Ccr2	NM_021866.1	ATTCTCTATTTCCAAGAAGTATCCAAGAGCTTGATGAGGGGGCCACCACA CCGTATGACTATGATGATGGTGAACCTTGTCATAAAACCAGTGTGAAGCA
Ccr5	NM_053960.3	AGGACATGAAGAGAATGGTCAAGGCCGAATCCTAAGGCATCATATCAGCA TTGCTTCTTCAGAGTTATGAGCATGGTGGGGAGCACCTAGACAGGTTTGT
Cd68 (ED1)	NM_001031638.1	CTCTCATTCCCTTACGGACAGCTTACCTTTGGATTCAAACAGGACCGACA TCAGAGCCACAGTACAGTCTACCTTAACTACATGGCAGTGGAATACAATG
Cd163	NM_001107887.1	CCTCTGTAATTTGCTCAGGAAACCAATCGCATACACTGTTGCCATGTAGT TCATCATCTTCGGTCCAAACAACAAGTTCTACCATTGCAAAGGACAGTGA
Chi3l3 (YM1)	NM_001191712.1	AAGCGTTTGAGAAAGAATCTACTGAGCAAGAAATCCCAAGGCTGCTTCTC ACTGCCACAGTAGCTGGAGTCATTGACACAATCCAGTCTGGTTACAAGAT
Csf1r	NM_001029901.1	ACCTACCTAGGGCCGTTCTTTGAAGACCCACACAATCTTGAGTTTAGAAC CCAATGGACCACATACAGCTACTCATTCAAACTCCACCTGAACCGTGTAA
Cx3cr1	NM_133534.1	ATGTGCAAGCTCACGACTGCTTTCTTCTTCATTGGCTTCTTTGGGGGCAT ATTCTTCATCACCGTCATCAGCATCGACCGGTACCTCGCCATCGTCCTGG
Cybb (NOX2)	NM_023965.1	CAGTACCAAAGTTTGCCGGAAACCCTCCTATGACTTGGAAATGGATCGTG GGTCCCATGTTCCTGTATCTGTGTGAGAGGCTGGTGCGGTTTTGGCGATC
Fcgr1a	NM_001100836.1	TGATGGATCATACTGGTGCGAGGTAGCCACGGAGGACGGCCGTGTCCTTA AGCGCAGCACCAAGTTGGAGCTATTTGGTCCCCAGTCATCAGATCCTGTC
Fcgr2b	NM_175756.1	CTGGTCCAAGGAATGCTGTAGATATGAAAGAAAACATCTAGAGTCCCTTC

		TGTGAGTCCTGAAACCAACAGACACTACGATATTGGTTCCCAATGGTTGA
Fcgr3a	NM_207603.1	GACTCTTGTTTGCAATAGACACAGTGCTGTATTTCTCGGTGCAGAGGAGT CTTCAAAGTTCCGTGGCAGTCTATGAGGAACCCAAACTTCACTGGAGCAA
Gusb	NM_017015.2	TCATTTGATCCTGGATGAGAAACGAAAAGAATATGTCATCGGAGAGCTCA TCTGGAATTTTGCTGACTTCATGACGAACCAGTCACCACTGAGAGTAACA
Havcr2 (TIM-3)	NM_001100762.1	CGATGAAATTAAGGACTCTGGAGAAACTATCAGAACTGCTGTCCACATTG GAGTAGGCGTCTCTGCTGGGCTGGCCCTGGCACTTATTCTTGGTGTTTTA
Hprt1	NM_012583.2	AGCTTCCTCCTCAGACCGCTTTTCCCGCGAGCCGACCGGTTCTGTCATGT CGACCCTCAGTCCCAGCGTCGTGATTAGTGATGATGAACCAGGTTATGAC
Hvcn1	XM_006249369.2	ACCAAGAGGATGAGCAGGTTCTTGAAGCACTTCACAGTGGTGGGGGACGA CTACCACACCTGGAATGTCAACTACAAGAAGTGGGAGAACGAGGAGGATG
Ifng	NM_138880.2	AAGGACGGTAACACGAAAATACTTGAGAGCCAGATTATCTCTTTCTACCT CAGACTCTTTGAAGTCTTGAAAGACAACCAGGCCATCAGCAACAACATAA
Ifngr1	NM_053783.1	CCTGTTACACATTCGACTACACTGTGTTTGTGAAACATTACAGGAGTGGG GAGATCCTACATACAGAACATAGCGTCCTAAAAGAAGATTGTAGCGAAAC
Ifngr2	NM_001108313.1	TTTCTTAAGTTACACTTAGTAAAGCAGATGAGTCCGCAGGAGACTTCAGC AAGAAAGAAGTTCCTACCGTCTCATCCCTTAGTTCTTCAAAGCCAAAGGA
Il1b	NM_031512.1	TGCACTGCAGGCTTCGAGATGAACAACAAAAATGCCTCGTGCTGTCTGAC CCATGTGAGCTGAAAGCTCTCCACCTCAATGGACAGAACATAAGCCAACA
Il1r1	NM_013123.3	CTCATATTCTGGAGACTGCACACGTACGGTTAGTATACCCAGTTCCTGAC TTCAAGAATTACCTCATCGGGGGCTTTGCCATCTTCACAGCTACAGCCGT
Il1r2	NM_053953.1	CCACTGTGAACAAATGTCTCTGGAACTCAAGGTCTTTAAGAATACCGAAG CCTCTTTCCCTCTCGTCTCCTACTTGCAAATCTCAGCTCTCTCCTCCACC
Il1rn	NM_022194.2	TCATTGCTGGGTACTTACAAGGACCAAATACCAAACTAGAAGAAAAGATA GACATGGTGCCTATTGACTTTCGGAATGTGTTCTTGGGCATCCACGGGGG
Il4	NM_201270.1	TGCTGTCACCCTGTTCTGCTTTCTCATATGTACCGGGAACGGTATCCACG GATGTAACGACAGCCCTCTGAGAGAGATCATCAACACTTTGAACCAGGTC
Il4r	NM_133380.2	GGGTGTCAGCATCTCCTGCATCTGCATCCTATTGTTTTGCCTGACCTGTT ACTTCAGCATTATCAAGATTAAGAAGATATGGTGGGACCAGATTCCCACT
Il6	NM_012589.1	GGAACAGCTATGAAGTTTCTCTCCGCAAGAGACTTCCAGCCAGTTGCCTT CTTGGGACTGATGTTGTTGACAGCCACTGCCTTCCCTACTTCACAAGTCC
Il10	NM_012854.2	ACAACATACTGCTGACAGATTCCTTACTGCAGGACTTTAAGGGTTACTTG GGTTGCCAAGCCTTGTCAGAAATGATCAAGTTTTACCTGGTAGAAGTGAT
Il10ra	NM_057193.2	TGTTTACATGTCACGACGGAGCATTATTTCACCGTGACCAACCTCAGCAT TTTCTTCTTATCCATCCTGATACTCTGTGGAGCCCTGGTCTGCCTGGTTC
Il10rb	NM_001107111.1	CCTCCCTGGATCGTGGCCATCATCCTTATAGCCTCCGTCTTGATAGTCTT CCTCTTCCTACTGGGCTGCTTCAGCATGGTGTGGTTCATTTACAAGAAGA
Il13ra1	NM_145789.2	TAACGAATTTGAGTGTCTCTGTCGAAAATCTCTGCACAATAGTGTGGACA TGGAGTCCTCCTGAGGGAGCCAGTCCAAATTGCAGTCTCAGATATTTTAG
Itgam (Cd11b)	NM_012711.1	CATCCCTTCCTTCAACAGTAAAGAAATATTCAACGTCACCCTCCAGGGCA ATCTGCTATTTGACTGGTACATCGAGACTTCTCATGACCACCTCCTGCTT
Itgb2	NM_001037780.2	CAATATCAGGTGCAACGGAGTCAACTGTCTCCACAAAAAGTGACCCTTAA CTTGCGACCAGGGCAGGCTGCTGCATTCAATGTGACTTTCCGACGGGCCA
Kcna2 (Kv1.2)	NM_012970.3	GCCGGCCAGGATCATAGCCATTGTATCTGTGATGGTCATTCTGATCTCGA TCGTCAGCTTCTGTCTGGAAACCTTGCCCATCTTCCGGGATGAGAACGAG
Kcna3 (Kv1.3)	NM_019270.3	GCCACCTTCTCCAGAAATATCATGAACCTGATAGACATTGTAGCCATCAT CCCTTATTTTATTACTCTGGGCACTGAGCTGGCTGAGCGACAGGGTAATG
Kcna5 (Kv1.5)	NM_012972.1	ATCAGAAGGGGTAGCTGTCCTCTAGAAAAGTGTCACCTCAAGGCCAAGAG CAACGTGGACTTGCGGAGGTCCCTGTATGCCCTCTGTCTGGACACTAGCC
Kcnj2 (Kir2.1)	NM_017296.1	GTTCTTTGGCTGTGTGTTTTGGTTGATAGCTCTGCTCCACGGGGATCTGG ATGCTTCTAAAGAGAGCAAAGCGTGTGTGTCTGAGGTCAACAGCTTCACG
Kcnma1	NM_031828.1	TGATATCTGCCCAGACACTGACTGGCAGAGTCCTGGTTGTGTTAGTCTTT

(Bk)		GCTCTCAGCATTGGTGCCCTTGTAATATACTTCATAGACTCATCAAACCC
Kcnn3 (SK3)	NM_019315.2	AGAGAAAGCGACTGAGTGACTATGCTCTGATTTTTGGGATGTTTGGAATT GTTGTTATGGTGATAGAGACCGAACTGTCTTGGGGTTTGTACTCAAAGGA
Kcnn4 (SK4)	NM_023021.2	TACGTCTCTACCTGGTGCCTCGCGCGGTACTTCTGCGTAGCGGGGTCCTG CTCAACGCGTCTTACCGCAGCATCGGGGCGCTCAACCAAGTCCGATTCCG
Kdm6b (JMJD3)	NM_001108829.1	CTGCGATCGGCATGGTGTGGATTACTTGACTGGTTCCTGGTGGCCAATCT TGGATGACCTCTATGCGTCCAATATTCCTGTTTACCGCTTCGTGCAGCGC
Mrc1 (CD206)	NM_001106123.1	CTTTGGAATCAAGGGCACAGAGCTATATTTTAACTATGGCAACAGGCAAG AAAAGAATATCAAGCTTTACAAAGGTTCCGGTTTGTGGAGCAGATGGAAG
Msr1 (SR-A)	NM_001191939.1	CACGTTCCATGACAGCATCCCTTCCTCACAACACTATAAATGGCTCCTCC GTTCAGGAGAAACTGAAGTCCTTCAAAGTTGCCCTCGTCGCTCTCTACCT
Mtmr6	NM_001107268.1	CTTGACCGGTTTAGTACCAACAACAAGTCACTGACAGGAACACTGTATCT TACGGCCACACACCTATTATTTATTGATGCTCATCAAAAAGAAACCTGGA
Myc	NM_012603.2	ACCGAGGAAAACGACAAGAGGCGGACACACAACGTCTTGGAACGTCAGAG GAGAAACGAGCTGAAGCGTAGCTTTTTTGCCCTGCGCGACCAGATCCCTG
Ncf1	NM_053734.2	TCCATTCCCAGCATCCCATAATTGGGCTTGTCCGTGTTCCAACATCTGGG CGGAATTTCACAGCCAAAGGTCAAGAGGACTGCTGTTACGTTCAAGGTCG
Nfkbia (IκBα)	NM_001105720.2	TATTGTGCTTTTGGTTGAACCGCCATAGACTGTAGCTGACCCCAGTGTGC CCTCTCACGTAAGAACCAGGTGTTCAGTGGTATGTGCTTAAGTCATCCCC
Nme2 (NDPK-B)	NM_031833.2	TGATTCAGTGGAGAGTGCCGAGAAAGAGATCGGTCTATGGTTTAAGCCCG AAGAACTGATTGACTATAAGTCTTGTGCCCATGACTGGGTGTATGAGTAG
Nos2	NM_012611.2	ACGGGACACAGTGTCGCTGGTTTGAAACTTCTCAGCCACCTTGGTGAGGG GACTGGACTTTTAGAGACGCTTCTGAGGTTCCTCAGGCTTGGGTCTTGTT
Nox1	NM_053683.1	CCGAGAAAGAAGATTCTTGGCTAAATCCCATCCAGTCTCCAAACGTGACA GTGATGTATGCAGCATTTACCAGTATTGCTGGCCTTACTGGAGTGGTCGC
Nox4	NM_053524.1	TGTTGGACAAAAGCAAGACTCTACATATCACCTGTGGCATAACTATTTGT ATTTTCTCAGGTGTGCATGTAGCTGCCCACTTGGTGAACGCCCTGAACTT
Nr3c1 (GR)	NM_012576.2	AGCTTTCCTTGAAGCGTATAAAGAGCCATGCTCCTTTAGTATGTGGGGAA GAAGAGAGCTGTCATAGTTTTGAGTACAGTGAGAAGATGCGGTACTGTCT
Orai1	NM_001013982.1	GCCTTCTCCACCGTCATCGGGACGCTGCTTTTCCTGGCCGAAGTCGTGCT GCTCTGCTGGGTGAAGTTCTTACCGCTCAAGAGGCAGGCGGGACAGCCAA
Orai3	NM_001014024.1	ACCTGTAATGTGCTTTACAGTTGGCATCCTGGGAGAGATTTTACATAGGC TCCTCAGATGAACCACTTTACACTTGGTGACTTGTGGTGGTGTGTCCCAC
P2rx7	NM_019256.1	ACTTTAAGAGGTCACATTAACCAGACTAGAAGCCATCGCATCTAACCGCA TACCAGACACAGTCTGACGCCTCATTGCTATGCTATGGTTCTAAGTGACT
P2ry2	NM_017255.1	GAGCTCTTTAGCCATTTTGTGGCTTACAGCTCTGTCATGCTGGGTCTGCT TTTTGCTGTGCCCTTTTCCATCATCCTGGTCTGTTACGTGCTCATGGCCC
P2ry6	NM_057124.2	TGGCCCAACATGCCTGGCCCTCCAAAATTTCTATGTCAACCACAAAACTA AGACACCTGTGTTTCGGGGACTGGTCAGTTCATGCTTGTTATACCAGAAT
P2ry12	NM_022800.1	TGATAACCATTGACCGATACCTGAAGACCACCAGACCATTTAAAACTTCC AGCCCCAGCAATCTTTTGGGTGCGAAGATTCTTTCTGTTGCCATCTGGGC
Phtp1	NM_001106558.2	CCTGTGTTCTCCTTTTGTACCTTGGGCAACACACCACCTGCCAGGCCTTA GAGGCCAGAGCAATCTGATCCATAGGAATTAAAGTATTGATATGCCTACT
Pparg	NM_013124.1	TTTATAGCTGTCATTATTCTCAGTGGAGACCGCCCAGGCTTGCTGAACGT GAAGCCCATCGAGGACATCCAAGACAACCTGCTGCAGGCCCTGGAACTCC
Prkaa1 (AMPK)	NM_019142.1	CACCAGAAGTAATTTCAGGAAGATTGTACGCAGGCCCTGAAGTAGACATC TGGAGCAGCGGGGTCATTCTCTATGCTTTGCTGTGTGGAACTCTCCCTTT
Ptgs2 (COX-2)	NM_017232.3	TTCGGAGGAGAAGTGGGTTTTAGGATCATCAACACTGCCTCAATTCAGTC TCTCATCTGCAATAATGTGAAAGGGTGTCCCTTTGCCTCTTTCAATGTGC
Ptk2b (PYK2)	NM_017318.2	GCAGTGATCATGAAGAATCTTGACCACCCTCACATCGTCAAGCTGATTGG CATCATTGAAGAGGAACCCACATGGATCGTCATGGAACTGTATCCTTATG
Ptpn6	NM_053908.1	GCAGAGTCACTGCTGCAGGCCAAGGGCGAGCCCTGGACATTTCTTGTGCG

(SHP-1)		TGAGAGTCTCAGCCAACCTGGTGATTTTGTGCTCTCTGTGCTCAATGACC
Rest	NM_031788.1	GCTGAGCTGGCTGCTCCCATGGAATCTACCAGTGCTTTATCCTCTGAACA<br>AAGCTCAAATGCACCAGATGGTGAAACATTACACAGCGAGTGTCAGGCTG
Retnla (Fizz1)	NM_053333.1	AGGAACTTCTAGCCCATCAAGATAACTATCCCTCTGCTGTAAGGAAGACC<br>CTCTCATGCACTAATGTCAAGTCTATGAGCAAATGGGCCTCCTGCCCTGC
Sirpa	NM_013016.2	AGGACATTCATTCTCGGGTCATCTGCGAGGTAGCCCACGTCACCTTGGAA<br>GGACGCCCGCTTAATGGGACCGCTAACTTTTCTAACATCATCCGAGTTTC
Slc8a1	NM_001270772.1	AGAGATCGAACAATTAATAGAATTAGCCAACTATCAAGTCCTAAGTCAGC<br>AGCAAAAGAGCCGAGCATTTTACCGAATTCAAGCTACTCGCCTGATGACT
Socs1	NM_145879.1	CGGCCGCTGCAGGAGCTGTGTCGCCAGCGCATCGTGGCCGCCGTGGGTCG<br>CGAGAACCTGGCACGCATCCCTCTTAACCCGGTACTCCGTGACTACCTGA
Socs3	NM_053565.1	GGAAGACTGTCAACGGTCACCTGGACTCCTATGAGAAAGTGACCCAGCTG<br>CCTGGACCCATTCGGGAGTTCCTGGACCAGTATGATGCTCCACTTTAAAG
Sparc	NM_012656.1	TGTTGGTTTTAATTTTGGTGAGCCAAGGGGAGGCATGGGCAGACCAATAC<br>CTCACTAGGGATTCTCTTACTCAACTGCTATAGGGCTTTCAGGCTCTTGC
Stim1	NM_001108496.2	TATCTATCGTGATTGGTGTGGGTGGCTGCTGGTTTGCCTATATCCAGAAC<br>CGTTACTCTAAGGAGCACATGAAGAAAATGATGAAGGATCTGGAAGGATT
Stim2	NM_001105750.2	TTCACAATTGGACGCTTGAGGATACCCTGCAGTGGTTGATAGAATTTGTT<br>GAACTCCCACAATACGAGAAGAATTTTAGGGATAATAATGTGAAAGGAAC
Tgfb1	NM_021578.2	CGCCTGCAGAGATTCAAGTCAACTGTGGAGCAACACGTAGAACTCTACCA<br>GAAATATAGCAACAATTCCTGGCGTTACCTTGGTAACCGGCTGCTGACCC
Tgfbr1	NM_012775.2	GTCTGCATTGCACTTATGCTGATGGTCTATATCTGCCATAACCGCACTGT<br>CATTCACCACCGCGTACCAAATGAAGAGGATCCCTCACTAGATCGCCCTT
Tgfbr2	NM_031132.3	CCAGCAGTCCTGACCTGTTGCTGGTCATTATCCAAGTGACGGGCGTCAGC<br>CTCCTGCCTCCGCTGGGGATTGCCATAGCTGTCATTGCCATCTTCTACTG
Tlr2	NM_198769.2	TTTACAAACCCTTAGGGTAGGAAATGTTGACACTTTCAGTGAGATAAGGA<br>GAATAGATTTTGCTGGGCTGACCTCTCTCAACGAACTTGAAATTCAGGTA
Tlr4	NM_019178.1	GTCAGTGTGCTTGTGGTAGCCACTGTAGCATTTCTGATATACCACTTCTA<br>TTTTCACCTGATACTTATTGCTGGCTGTAAAAAGTACAGCAGAGGAGAAA
Tnf	NM_012675.2	GGTGATCGGTCCCAACAAGGAGGAGAAGTTCCCAAATGGGCTCCCTCTCA<br>TCAGTTCCATGGCCCAGACCCTCACACTCAGATCATCTTCTCAAAACTCG
Tnfrsf1a (TNFR1)	NM_013091.1	TATTCTTTATCTGCATCAGTCTACTGTGCCGATATCCCCAGTGGAGGCCC<br>AGGGTCTACTCCATCATTTGTAGGGATTCAGCTCCTGTCAAAGAGGTGGA
Tnfrsf1b (TNFR2)	NM_130426.4	AGGAGTTCAGATTCTTCCCATGGCAGCCACGGGACCCATGTCAACGTCAC<br>CTGCATCGTGAACGTCTGTAGCAGCTCTGACCACAGCTCTCAGTGTTCTT
Trem1	NM_001106885.1	TCAAATGACTGACCTTCAAGTGACAGACTCTGGATTATATCGTTGTGTGA<br>TTTACCATCCTCCGAACGACCCTGTTCTGCTCTTCCATCCCGTCCGCCTG
Trem2	NM_001106884.1	TCCGGCTGGCTGAGGAAGGGTGCCATGGAACCTCTCCACGTGTTTGTCCT<br>GTTGCTGGTCACAGAGCTGTCCCAAGCCCTCAACACCACAGTGCTGCAGG
Trpm2	NM_001011559.1	GTGAGCCTCCGGTCTCTCTATAAGCGATCAACAGGCCACGTTACCTTCAC<br>CATTGACCCAGTCCGCGATCTTCTCATTTGGGCCATCATCCAGAACCACA
Trpm4	NM_001136229.1	AACTATTCTGCTTTCTTCTTGGTGGATGATGGTACCTATGGCCGCATGGG<br>TGGTGAGAACCGCTTCCGCCTTCGGTTTGAGTCCTATGTGGCTCAGCAGA
Trpm7	XM_001056331.1	TTCTCTTCATTCAGAAACAGAGAGCTGTAGTAGAAGAGCGTCGACAGAAG<br>ACTCTCCGGACGTAGATTCCAGAGCAGCTTTGTTGCCGGATTGGTTACGA
Tspo	NM_012515.1	GCTGCCCGCTTGCTGTATCCTTACCTGGCCTGGCTGGCCTTTGCCACCAT<br>GCTCAACTACTATGTATGGCGTGATAACTCTGGTCGGCGAGGGGGCTCCC
