# Supplementary material for: Microglia Responses to Pro-inflammatory Stimuli (LPS, IFNγ+TNFα) and Reprogramming by Resolving Cytokines (IL-4, IL-10)
Source: Front Cell Neurosci. 2018 Jul 24;12:215. doi: 10.3389/fncel.2018.00215 (PMC6066613; doi:10.3389/fncel.2018.00215)
Supplement: Supplementary file 3 [file Table_3.pdf]

Microglia responses to pro-inflammatory stimuli (LPS, IFNγ + TNFα) and reprogramming by resolving cytokines (IL-4, IL-10)

Starlee Lively and Lyanne C. Schlichter*

* Correspondence: Professor Lyanne C. Schlichter Lyanne.Schlichter@uhnresearch.ca

Supplementary Table 3. Expression profile of cultured neonatal rat microglia 6 h after LPS or I+T treatment. Concentration is the same as for 24 h. Basal expression of unstimulated (Control) is expressed as mean counts ± SD (*n*=3–5 individual cultures). Effects of LPS or I+T are shown as fold-changes where arrows reflect increases (↑) or decreases (↓) relative to Control. Asterisks (*) indicate differences between LPS and I+T. For both, one symbol indicates $p<0.05$; two, $p<0.01$; three, $p<0.001$.

	Relative mRNA ***Counts*** *± SD*	***Fold change*** *with respect to Control* ± SD	
Gene	Control	LPS	I+T
**Pro-inflammatory Mediators**			
Casp1 (ICE)	818 ± 199	1.20 ± 1.23	0.90 ± 0.06
Il1b	315 ± 154	658.16 ± 669.01 ↑↑↑ ***	11.02 ± 1.32 ↑↑↑
Il1r2	10 ± 5	1.34 ± 1.64	1.27 ± 0.59
Il6	7 ± 3	6221.92 ± 6326.57 ↑↑↑ ***	21.78 ± 6.27 ↑↑↑
Nos2 (iNOS)	5 ± 5	14635.76 ± 14700.78 ↑↑↑	6355.92 ± 2156.46 ↑↑↑
Tnfa	428 ± 132	46.95 ± 48.70 ↑↑↑ ***	15.38 ± 1.36 ↑↑↑
**Anti-inflammatory Mediators**			
Arg1	4 ± 3	2.46 ± 2.77	2.14 ± 1.37
Ccl22	28 ± 4	14.74 ± 15.52 ↑↑↑ ***	2.21 ± 0.54 ↑↑
Cd163	4 ± 2	7.58 ± 8.25 ↑↑	1.91 ± 1.29
Chi3l3 (YM1)	23 ± 3	0.78 ± 0.84	0.62 ± 0.62
Il1rn (IL-1RA)	174 ± 26	54.83 ± 62.96 ↑↑↑ ***	4.21 ± 0.17 ↑↑↑
Il4	3 ± 2	1.43 ± 1.60	1.73 ± 0.97
Il4r	936 ± 25	1.10 ± 1.07	1.40 ± 0.10 ↑↑ *
Il10	2 ± 1	13.71 ± 15.17 ↑↑ *	2.69 ± 2.45
Il13ra1	477 ± 32	0.89 ± 0.88	1.70 ± 0.15 ↑↑↑ ***
Mrc1 (CD206)	2245 ± 283	0.20 ± 0.20 ↓↓↓	0.23 ± 0.08 ↓↓↓
Myc	437 ± 69	2.39 ± 2.21 ↑↑↑ ***	0.53 ± 0.09 ↓↓
Pparg	60 ± 24	0.55 ± 0.54	0.22 ± 0.14 ↓↓
Retnla (FIZZ1)	3 ± 2	4.22 ± 5.44	4.94 ± 1.34
Tgfb1	7135 ± 963	1.50 ± 1.49	1.09 ± 0.22
**Microglia Markers and Modulators**			
Cd68	19756 ± 1535	0.82 ±0.87	0.94 ± 0.01
Cx3cr1	994 ± 239	0.30 ± 0.27 ↓↓	0.02 ± 0.01 ↓↓↓ ***
Itgam (CD11b)	1407 ± 159	1.69 ± 1.77 ↑↑↑	2.00 ± 0.07 ↑↑↑
Tlr2	2490 ± 232	4.58 ± 4.62 ↑↑↑ ***	1.62 ± 0.12 ↑↑↑
Tlr4	714 ± 78	0.17 ± 0.17 ↓↓↓ *	0.39 ± 0.12 ↓↓
**Physiology-related**			
Ncf1	2891 ± 389	2.40 ± 2.43 ↑↑↑	7.51 ± 1.19 ↑↑↑ ***
P2rx7	55 ± 15	0.60 ± 0.61	1.64 ± 0.77 *
P2ry2	23 ± 2	10.32 ± 10.86 ↑↑↑ ***	2.99 ± 1.12 ↑↑
P2ry6	31 ± 7	0.83 ± 0.75	0.23 ± 0.09 ↓↓↓ **
**Ion channels and their regulators**			

Kcna3	83 ± 28		6.42 ± 7.00 ↑↑↑	8.98 ± 1.38 ↑↑↑
Kcna5	3 ± 2		3.63 ± 3.47	1.92 ± 1.51
Kcnj2	780 ± 154		6.50 ± 6.67 ↑↑↑ ***	2.05 ± 0.23 ↑↑↑
Kcnn3	7 ± 2		4.25 ± 4.86 ↑	1.54 ± 1.03
Kcnn4	49 ± 13		1.86 ± 1.87	1.69 ± 0.56
