# Supplementary material for: Microglia Responses to Pro-inflammatory Stimuli (LPS, IFNγ+TNFα) and Reprogramming by Resolving Cytokines (IL-4, IL-10)
Source: Front Cell Neurosci. 2018 Jul 24;12:215. doi: 10.3389/fncel.2018.00215 (PMC6066613; doi:10.3389/fncel.2018.00215)
Supplement: Supplementary file 4 [file Table_4.pdf]

Microglia responses to pro-inflammatory stimuli (LPS, IFNγ + TNFα) and reprogramming by resolving cytokines (IL-4, IL-10)

Starlee Lively and Lyanne C. Schlichter*

* Correspondence: Professor Lyanne C. Schlichter Lyanne.Schlichter@uhnresearch.ca

Supplementary Table 4. Repolarization by IL-4 or IL-10: Pro-inflammatory genes. Rat microglia were stimulated with LPS or IFNγ + TNFα (I+T) and 2 h later, IL-4 or IL-10 was added for a further 22 h. Results are shown as fold changes (mean ± SD). Arrows indicate statistical differences from unstimulated control cells; while arrowheads show effects of IL-4 or IL-10 on LPS- or I+T-treated cells (decreases in red; increases in blue). n=6–7 individual cultures for every condition. Results were analyzed by 1-way ANOVA (with Tukey's test); one symbol of any type indicates $p<0.05$; two, $p<0.01$; three, $p<0.001$.

Gene	*Fold change with respect to Control*					
	LPS	+IL-4	+IL-10	I+T	+IL-4	+IL-10
C1r	4.15 ± 2.39 ↑↑	1.98 ± 0.84	6.81 ± 2.85 ↑↑↑	5.54 ± 3.35 ↑↑↑	1.29 ± 0.85 ▼▼	6.06 ± 3.27 ↑↑↑
C5ar	6.86 ± 1.32 ↑↑↑	0.98 ± 0.21 ▼▼▼	5.03 ± 1.00 ↑↑↑	0.25 ± 0.07 ↓↓↓	0.10 ± 0.01 ↓↓↓ ▼▼▼	0.41 ± 0.13 ↓↓↓ ▲
Casp1 (ICE)	2.80 ± 0.72 ↑↑↑	0.99 ± 0.24 ▼▼▼	2.88 ± 0.57 ↑↑↑	1.55 ± 0.10 ↑	0.52 ± 0.03 ↓↓↓ ▼▼▼	1.90 ± 0.25 ↑↑↑
Ccl3	71.68 ± 16.04 ↑↑↑	48.48 ± 12.56 ↑↑↑	38.71 ± 10.78 ↑↑↑ ▼▼	0.52 ± 0.12 ↓↓	0.05 ± 0.01 ↓↓↓ ▼▼▼	0.65 ± 0.17 ↓
Ifng	4.17 ± 3.60 ↑	1.34 ± 0.94	1.28 ± 0.45	1.50 ±1.39	0.45 ± 0.23	0.84 ± 0.67
Ifngr1	1.71 ± 0.71	0.74 ± 0.19 ▼▼▼	1.50 ± 0.19	1.26 ± 0.26	0.21 ± 0.05 ↓↓↓ ▼▼▼	1.36 ± 0.31
Ifngr2	2.11 ± 0.50 ↑↑↑	2.19 ± 1.18	2.10 ± 0.90	1.52 ± 0.22 ↑	0.88 ± 0.11 ▼▼	2.08 ± 0.43 ↑↑↑
Il1b	192.60 ± 31.04 ↑↑↑	97.10 ± 29.97 ↑↑↑	104.73 ± 41.18 ↑↑↑	1.24 ± 0.33	0.10 ± 0.03 ↓↓↓ ▼▼▼	1.46 ± 0.53
Il1r1	14.79 ± 5.41 ↑↑↑	9.26 ± 2.70 ↑↑↑	8.33 ± 2.78 ↑↑↑	1.72 ± 0.48	0.49 ± 0.13 ↓ ▼▼▼	1.86 ± 0.67 ↑
Il1r2	7.79 ± 5.20 ↑↑↑	4.92 ± 2.95 ↑↑↑	2.79 ± 0.83 ↑↑↑	1.80 ± 0.75	0.32 ± 0.13 ↓ ▼▼▼	1.28 ± 0.67
Il6	2910.45 ± 649.57 ↑↑↑	3704.08 ± 1238.84 ↑↑↑	1309.72 ± 538.80 ↑↑↑	4.17 ± 2.23 ↑↑	1.55 ± 0.85	5.38 ± 1.05 ↑↑↑
Nos2 (iNOS)	5479.50 ± 1313.28 ↑↑↑	1809.47 ± 626.34 ↑↑↑ ▼	2658.24 ± 685.12 ↑↑↑	1030.13 ± 125.70 ↑↑↑	24.32 ± 22.81 ↑↑↑ ▼▼▼	1574.33 ± 313.60 ↑↑↑
Ptgs2 (COX-2)	1471.12 ± 710.39 ↑↑↑	636.96 ± 535.71 ↑↑↑	353.76 ± 170.63 ↑↑↑ ▼	28.66 ± 17.08 ↑↑↑	13.48 ± 5.22 ↑↑↑	43.86 ± 29.46 ↑↑↑
Ptk2b (PYK2)	6.28 ± 0.97 ↑↑↑	2.57 ± 0.39 ↑↑↑ ▼▼▼	7.77 ± 0.71 ↑↑↑	11.75 ± 0.43 ↑↑↑	2.51 ± 0.29 ↑↑↑ ▼▼▼	13.40 ± 1.06 ↑↑↑
Tnf	8.15 ± 1.60 ↑↑↑	2.11 ± 0.52 ↑↑ ▼▼▼	6.56 ± 2.13 ↑↑↑	4.20 ± 0.88 ↑↑↑	0.93 ± 0.11 ▼▼▼	4.23 ± 1.47 ↑↑↑
Tnfrsf1a (TNFR1)	3.38 ± 0.77 ↑↑↑	1.15 ± 0.26 ▼▼▼	3.11 ± 0.28 ↑↑↑	3.49 ± 0.43 ↑↑↑	0.76 ± 0.09 ↓↓ ▼▼▼	4.44 ± 0.75 ↑↑↑ ▲
Tnfrsf1b (TNFR2)	22.91 ± 4.37 ↑↑↑	22.86 ± 7.78 ↑↑↑	22.57 ± 3.70 ↑↑↑	2.71 ± 0.40 ↑↑↑	0.22 ± 0.03 ↓↓↓ ▼▼▼	3.61 ± 0.81 ↑↑↑
