# Supplementary material for: Microglia Responses to Pro-inflammatory Stimuli (LPS, IFNγ+TNFα) and Reprogramming by Resolving Cytokines (IL-4, IL-10)
Source: Front Cell Neurosci. 2018 Jul 24;12:215. doi: 10.3389/fncel.2018.00215 (PMC6066613; doi:10.3389/fncel.2018.00215)
Supplement: Supplementary file 5 [file Table_5.pdf]

Microglia responses to pro-inflammatory stimuli (LPS, IFNγ + TNFα) and reprogramming by resolving cytokines (IL-4, IL-10)

Starlee Lively and Lyanne C. Schlichter*

* Correspondence: Professor Lyanne C. Schlichter Lyanne.Schlichter@uhnresearch.ca

Supplementary Table 5. Repolarization: Anti-inflammatory genes and receptors. Rat microglia were stimulated with LPS or IFNγ + TNFα (I+T) and 2 h later, IL-4 or IL-10 was added for a further 22 h. Results are shown as fold changes (mean ± SD). Arrows indicate statistical differences from unstimulated control cells; while arrowheads show effects of IL-4 or IL-10 on LPS- or I+T-treated cells (decreases in red; increases in blue). n=6–7 individual cultures for every condition. Results were analyzed by 1-way ANOVA (with Tukey's test); one symbol of any type indicates $p<0.05$; two, $p<0.01$; three, $p<0.001$.

Gene	*Fold change with respect to Control* (± SD)					
	LPS	+IL-4	+IL-10	I+T	+IL-4	+IL-10
Arg1	1216.02 ± 419.03 ↑↑↑	4666.40 ± 2287.02 ↑↑↑ ▲▲	1156.45 ± 453.15 ↑↑↑	4.73 ± 3.00 ↑↑	0.96 ± 1.07 ▼	17.82 ± 16.59 ↑↑↑
Ccl22	81.39 ± 42.61 ↑↑↑	638.01 ± 548.96 ↑↑↑ ▲	34.91 ± ↑↑↑ 29.69	3.36 ± 0.70 ↑↑	228.45 ± 121.98 ↑↑↑ ▲▲▲	3.68 ± 0.92 ↑↑↑
Cd163	8.10 ± 3.85 ↑↑↑	5.43 ± 2.63 ↑↑↑	3.03 ± 2.04 ↑	1.69 ± 1.06	0.33 ± 0.23 ▼▼	1.50 ± 0.74
Chi3l3 (YM1)	3.46 ± 1.03 ↑↑	1.92 ± 1.32	2.34 ± 1.11	1.57 ± 0.84	0.22 ± 0.05 ↓↓ ▼▼▼	1.03 ± 0.31
Il1rn (IL-RA)	2.42 ± 1.58	0.49 ± 0.11 ▼▼▼	3.19 ± 0.52 ↑↑↑	3.37 ± 1.17 ↑↑	0.08 ± 0.03 ↓↓↓ ▼▼▼	4.59 ± 2.11 ↑↑↑
Il4	4.58 ± 3.40 ↑	0.77 ± 0.44 ▼▼	1.53 ± 1.09	1.04 ± 0.45	0.33 ± 0.18 ↓ ▼	1.11 ± 0.84
Il4r	10.16 ± 3.19 ↑↑↑	4.11 ± 1.25 ↑↑↑ ▼▼▼	11.56 ± 3.71 ↑↑↑	7.38 ± 0.99 ↑↑↑	1.80 ± 0.38 ↑↑↑ ▼▼▼	8.72 ± 2.11 ↑↑↑
Il10	36.88 ± 10.75 ↑↑↑	21.60 ± 17.39 ↑↑↑	40.65 ± 18.04 ↑↑↑	0.24 ± 0.16	0.11 ± 0.01 ↓	0.61 ± 0.42
Il10ra	2.09 ± 0.36 ↑↑↑	2.95 ± 0.63 ↑↑↑ ▲	2.89 ± 0.36 ↑↑↑ ▲	4.10 ± 0.59 ↑↑↑	1.28 ± 0.17 ▼▼▼	4.29 ± 0.98 ↑↑↑
Il10rb	3.18 ± 0.59 ↑↑↑	1.85 ± 0.65 ↑↑↑ ▼▼▼	2.79 ± 0.14 ↑↑↑	2.00 ± 0.13 ↑↑↑	0.47 ± 0.06 ↓↓↓ ▼▼▼	2.33 ± 0.29 ↑↑↑
Il13ra1	6.39 ± 1.36 ↑↑↑	1.65 ± 0.49 ↑↑↑ ▼▼▼	4.61 ± 1.01 ↑↑↑	2.57 ± 0.31 ↑↑↑	0.49 ± 0.04 ↓↓↓ ▼▼▼	3.84 ± 0.56 ↑↑↑ ▲▲▲
Mrc1 (CD206)	1.13 ± 0.40	4.92 ± 1.59 ↑↑↑ ▲▲▲	1.59 ± 0.39	0.03 ± 0.03 ↓↓↓	0.96 ± 0.11 ▲▲▲	0.02 ± 0.01 ↓↓↓
Myc	1.05 ± 0.33	3.27 ± 0.31 ↑↑↑ ▲▲▲	0.69 ± 0.14 ↓ ▼	0.24 ± 0.11 ↓↓↓	3.41 ± 0.42 ↑↑↑ ▲▲▲	0.23 ± 0.07 ↓↓↓
Pparg	0.14 ± 0.05 ↓↓↓	0.08 ± 0.05 ↓↓↓	0.07 ± 0.02 ↓↓↓	0.06 ± 0.03 ↓↓↓	0.04 ± 0.02 ↓↓↓	0.06 ± 0.01 ↓↓↓
Retnla (FIZZ1)	3.80 ± 1.29 ↑↑	1.93 ± 0.94	2.56 ± 1.21 ↑	1.60 ± 1.01	0.43 ± 0.16	1.63 ± 0.73

Tgfb1	1.18 ± 0.16	0.46 ± 0.06 ↓↓↓ ▼▼▼	1.14 ± 0.16		0.51 ± 0.07 ↓↓↓	0.37 ± 0.02 ↓↓↓ ▼▼▼	0.55 ± 0.05 ↓↓↓
Tgfbr1	0.73 ± 0.17 ↓	0.29 ± 0.06 ↓↓↓ ▼▼▼	0.54 ± 0.08 ↓↓↓		1.21 ± 0.21	0.30 ± 0.04 ↓↓↓ ▼▼▼	1.22 ± 0.33
Tgfbr2	3.37 ± 0.61 ↑↑↑	1.47 ± 0.40 ↑ ▼▼▼	3.74 ± 0.65 ↑↑↑		1.99 ± 0.04 ↑↑↑	1.20 ± 0.17 ↑ ▼▼▼	2.45 ± 0.20 ↑↑↑ ▲▲
