# Supplementary material for: Microglia Responses to Pro-inflammatory Stimuli (LPS, IFNγ+TNFα) and Reprogramming by Resolving Cytokines (IL-4, IL-10)
Source: Front Cell Neurosci. 2018 Jul 24;12:215. doi: 10.3389/fncel.2018.00215 (PMC6066613; doi:10.3389/fncel.2018.00215)
Supplement: Supplementary file 6 [file Table_6.pdf]

Microglia responses to pro-inflammatory stimuli (LPS, IFNγ + TNFα) and reprogramming by resolving cytokines (IL-4, IL-10)

Starlee Lively and Lyanne C. Schlichter*

* Correspondence: Professor Lyanne C. Schlichter Lyanne.Schlichter@uhnresearch.ca

Supplementary Table 6. Repolarization: Microglial activation markers and immune modulators. Rat microglia were stimulated with LPS or IFNγ + TNFα (I+T) and 2 h later, IL-4 or IL-10 was added for a further 22 h. Results are shown as fold changes (mean ± SD). Arrows indicate statistical differences from unstimulated control cells; while arrowheads show effects of IL-4 or IL-10 on LPS- or I+T-treated cells (decreases in red; increases in blue). n=6–7 individual cultures for every condition. Results were analyzed by 1-way ANOVA (with Tukey's test); one symbol of any type indicates $p<0.05$; two, $p<0.01$; three, $p<0.001$.

Gene	*Fold change with respect to Control* (± SD)					
	LPS	+IL-4	+IL-10	I+T	+IL-4	+IL-10
Ager (RAGE)	5.99 ± 2.13 ↑↑	3.43 ± 2.35 ↑	4.46 ± 3.88 ↑↑	0.84 ± 0.35	0.57 ± 0.06	1.64 ± 1.29
Aif (Iba1)	3.10 ± 0.50 ↑↑↑	1.39 ± 0.19 ↑↑ ▼▼▼	3.17 ± 0.37 ↑↑↑	1.78 ± 0.26 ↑↑↑	0.76 ± 0.08 ↓ ▼▼▼	1.72 ± 0.33 ↑↑↑
Ccr2	7.00 ± 3.82 ↑↑↑	2.70 ± 2.00	2.85 ± 1.61	1.05 ± 0.45	0.32 ± 0.19 ↓ ▼▼	1.46 ± 0.59
Ccr5	0.52 ± 0.25 ↓	0.55 ± 0.24	0.92 ± 0.26	2.33 ± 0.27 ↑↑	0.33 ± 0.08 ↓↓↓ ▼▼▼	2.18 ± 0.32
Cd68 (ED1)	0.89 ± 0.15	0.57 ± 0.12 ↓↓↓ ▼▼	1.19 ± 0.12	0.64 ± 0.07 ↓↓	0.29 ± 0.07 ↓↓↓ ▼▼▼	0.68 ± 0.11 ↓
Csf1r	0.66 ± 0.18 ↓↓	0.36 ± 0.12 ↓↓↓ ▼▼	0.97 ± 0.12 ▲	1.10 ± 0.07	0.31 ± 0.04 ↓↓ ▼▼▼	1.10 ± 0.08
Cx3cr1	0.12 ± 0.06 ↓↓↓	0.03 ± 0.01 ↓↓↓ ▼▼▼	0.09 ± 0.04 ↓↓↓	0.03 ± 0.01 ↓↓↓	0.02 ± 0.00 ↓↓↓	0.02 ± 0.01 ↓↓↓
Itgam (CD11b)	4.17 ± 0.62 ↑↑↑	1.10 ± 0.32 ▼▼▼	3.84 ± 0.55 ↑↑↑	1.21 ± 0.12	0.17 ± 0.06 ↓↓↓ ▼▼▼	1.60 ± 0.25 ↑↑
Kdm6b (JMJD3)	7.87 ± 1.31 ↑↑↑	4.16 ± 1.57 ↑↑↑ ▼▼▼	5.53 ± 1.28 ↑↑↑	2.90 ± 0.24 ↑↑↑	0.61 ± 0.10 ↓↓↓ ▼▼▼	3.70 ± 0.50 ↑↑↑
Nfkbia	14.55 ± 3.17 ↑↑↑	6.66 ± 1.64 ↑↑↑ ▼▼	11.37 ± 1.83 ↑↑↑	5.99 ± 0.40 ↑↑↑	1.50 ± 0.15 ↑ ▼▼▼	5.58 ± 0.45 ↑↑↑
Nr3c1 (GR)	2.16 ± 0.38 ↑↑↑	1.03 ± 0.19 ▼▼▼	1.85 ± 0.19 ↑↑↑	3.87 ± 0.41 ↑↑↑	1.03 ± 0.13 ▼▼▼	3.96 ± 0.59 ↑↑↑
Prkaa1 (AMPK)	3.48 ± 0.62 ↑↑↑	1.65 ± 0.38 ↑↑↑ ▼▼▼	2.47 ± 0.30 ↑↑↑ ▼	1.29 ± 0.18	0.64 ± 0.08 ↓↓↓ ▼▼▼	1.41 ± 0.22 ↑↑
Socs1	17.90 ± 3.12 ↑↑↑	74.55 ± 22.84 ↑↑↑ ▲▲▲	10.14 ± 4.72 ↑↑↑ ▼	121.09 ± 34.67 ↑↑↑	88.07 ± 10.91 ↑↑↑	157.53 ± 60.61 ↑↑↑
Socs3	226.86 ± 52.43 ↑↑↑	140.15 ± 46.67 ↑↑↑	215.76 ± 80.81 ↑↑↑	12.09 ± 4.61 ↑↑↑	3.20 ± 0.71 ↑↑↑ ▼▼▼	19.23 ± 9.93 ↑↑↑
Sparc	0.26 ± 0.06 ↓↓↓	0.11 ± 0.04 ↓↓↓ ▼▼▼	0.36 ± 0.05 ↓↓↓	0.21 ± 0.08 ↓↓↓	0.06 ± 0.02 ↓↓↓ ▼▼▼	0.27 ± 0.12 ↓↓↓

Tlr2	5.30 ± 0.80 ↑↑↑	2.23 ± 0.72 ↑↑↑ ▼▼▼	4.74 ± 0.45 ↑↑↑		1.30 ± 0.32	0.11 ± 0.03 ↓↓↓ ▼▼▼	1.45 ± 0.22
Tlr4	1.31 ± 0.24	3.00 ± 1.01 ↑↑↑ ▲▲▲	1.29 ± 0.18		0.66 ± 0.16 ↓	0.50 ± 0.07 ↓↓↓	0.73 ± 0.19
Trem1	47.7 ± 17.1 ↑↑↑	7.6 ± 2.9 ↑↑↑ ▼▼▼	39.5 ± 19.1 ↑↑↑		1.4 ± 0.4	0.1 ± 0.0 ↓↓↓ ▼▼▼	3.0 ± 1.2 ↑↑↑
Trem2	0.38 ± 0.09 ↓↓↓	0.19 ± 0.04 ↓↓↓ ▼▼▼	0.44 ± 0.07 ↓↓↓		0.05 ± 0.02 ↓↓↓	0.02 ± 0.01 ↓↓↓ ▼▼▼	0.04 ± 0.01 ↓↓↓
Tspo	4.41 ± 1.24 ↑↑↑	0.79 ± 0.20 ▼▼▼	6.14 ± 0.43 ↑↑↑		3.10 ± 0.54 ↑↑↑	0.68 ± 0.12 ▼▼▼	5.15 ± 1.05 ↑↑↑ ▲
