# Supplementary material for: Microglia Responses to Pro-inflammatory Stimuli (LPS, IFNγ+TNFα) and Reprogramming by Resolving Cytokines (IL-4, IL-10)
Source: Front Cell Neurosci. 2018 Jul 24;12:215. doi: 10.3389/fncel.2018.00215 (PMC6066613; doi:10.3389/fncel.2018.00215)
Supplement: Supplementary file 7 [file Table_7.pdf]

Microglia responses to pro-inflammatory stimuli (LPS, IFNγ + TNFα) and reprogramming by resolving cytokines (IL-4, IL-10)

Starlee Lively and Lyanne C. Schlichter*

* Correspondence: Professor Lyanne C. Schlichter Lyanne.Schlichter@uhnresearch.ca

Supplementary Table 7. Repolarization: Genes related to microglial physiological functions. Rat microglia were stimulated with LPS or IFNγ + TNFα (I+T) and 2 h later, IL-4 or IL-10 was added for a further 22 h. Results are shown as fold changes (mean ± SD). Arrows indicate statistical differences from unstimulated control cells; while arrowheads show effects of IL-4 or IL-10 on LPS- or I+T-treated cells (decreases in red; increases in blue). n=6–7 individual cultures for every condition. Results were analyzed by 1-way ANOVA (with Tukey's test); one symbol of any type indicates p<0.05; two, p<0.01; three, p<0.001.

	Fold change with respect to Control (± SD)					
Gene	LPS	+IL-4	+IL-10	I+T	+IL-4	+IL-10
Adora1	52.05 ± 10.24 ↑↑↑	11.20 ± 6.62 ↑↑↑ ▼▼▼	34.49 ± 13.57 ↑↑↑	2.56 ± 1.35 ↑	1.20 ± 0.72	3.69 ± 0.88 ↑↑
Adora2a	54.90 ± 11.80 ↑↑↑	53.67 ± 19.79 ↑↑↑	56.36 ± 8.46 ↑↑↑	22.56 ± 2.51 ↑↑↑	0.36 ± 0.19 ▼▼▼	33.42 ± 5.69 ↑↑↑
Axl	0.27 ± 0.08 ↓↓↓	0.14 ± 0.04 ↓↓↓ ▼▼	0.36 ± 0.08 ↓↓↓	0.29 ± 0.08 ↓↓↓	0.15 ± 0.06 ↓↓↓ ▼▼	0.29 ± 0.06 ↓↓↓
Cybb (NOX2)	1.12 ± 0.45	0.36 ± 0.11 ↓↓↓ ▼▼▼	2.34 ± 0.17 ↑↑↑ ▲▲▲	2.08 ± 0.29 ↑↑	0.23 ± 0.03 ↓↓↓ ▼▼▼	2.29 ± 0.36 ↑↑↑
Fcgr1a (CD64)	3.16 ± 0.95 ↑↑↑	0.30 ± 0.08 ↓↓↓ ▼▼▼	3.47 ± 0.62 ↑↑↑	0.46 ± 0.06 ↓↓	0.21 ± 0.03 ↓↓↓ ▼▼▼	0.56 ± 0.08 ↓↓
Fcgr2b (CD32B)	4.74 ± 1.11 ↑↑↑	16.92 ± 3.50 ↑↑↑ ▲▲▲	5.91 ± 1.78 ↑↑↑	0.80 ± 0.33	0.93 ± 0.12	1.25 ± 0.54
Fcgr3a (CD16A)	3.87 ± 1.08 ↑↑↑	2.06 ± 0.57 ↑↑	7.95 ± 0.95 ↑↑↑ ▲	4.12 ± 0.56 ↑↑↑	0.36 ± 0.08 ↓↓↓ ▼▼▼	6.23 ± 1.54 ↑↑↑
Havcr2 (TIM-3)	28.11 ± 12.4 ↑↑↑	7.39 ± 4.20 ↑↑↑ ▼▼▼	9.96 ± 3.74 ↑↑↑ ▼▼	3.29 ± 0.73 ↑↑↑	0.95 ± 0.19 ▼▼▼	7.06 ± 2.33 ↑↑↑ ▲▲▲
Hvcn1 (Hv1)	1.27 ± 0.31	0.44 ± 0.11 ↓↓↓ ▼▼▼	1.82 ± 0.35 ↑↑↑ ▲	2.81 ± 0.35 ↑↑↑	0.30 ± 0.06 ↓↓↓ ▼▼▼	2.47 ± 0.22 ↑↑↑
Itgb2	3.17 ± 0.46 ↑↑↑	2.25 ± 0.64 ↑↑↑ ▼	2.67 ± 0.47 ↑↑↑	0.62 ± 0.08 ↓↓↓	0.35 ± 0.08 ↓↓↓ ▼▼▼	0.77 ± 0.17
Msr1 (SR-A/CD204)	5.94 ± 0.91 ↑↑↑	1.21 ± 0.48 ▼▼▼	4.89 ± 1.39 ↑↑↑	0.12 ± 0.03 ↓↓↓	0.01 ± 0.01 ↓↓↓ ▼▼▼	0.17 ± 0.02 ↓↓↓
Ncf1 (p47phox)	5.43 ± 0.72 ↑↑↑	3.02 ± 0.51 ↑↑↑ ▼▼▼	5.31 ± 0.68 ↑↑↑	6.27 ± 1.34 ↑↑↑	1.50 ± 0.37 ↑ ▼▼▼	4.83 ± 0.70 ↑↑↑
Nox1	1.76 ± 0.97	0.61 ± 0.26 ▼	1.63 ± 1.32	0.53 ± 0.39	0.12 ± 0.00 ↓↓↓ ▼	0.66 ± 0.56
Nox4	15.84 ± 6.00 ↑↑↑	6.86 ± 3.88 ↑↑↑ ▼	12.26 ± 8.46 ↑↑↑	3.14 ± 2.02 ↑	1.01 ± 0.20 ▼	5.62 ± 3.07 ↑↑↑
P2rx7	0.32 ± 0.19 ↓↓	0.19 ± 0.02 ↓↓↓	0.20 ± 0.14 ↓↓↓	1.17 ± 0.55	1.26 ± 0.33	1.21 ± 0.66

P2ry2	15.58 ± 2.35<br>↑↑↑	21.90 ± 7.69<br>↑↑↑	14.99 ± 3.09<br>↑↑↑		4.62 ± 1.24<br>↑↑↑	2.41 ± 0.44<br>↑↑↑ ▼▼	7.14 ± 2.19<br>↑↑↑ ▲
P2ry6	5.43 ± 0.65<br>↑↑↑	6.89 ± 1.64<br>↑↑↑	8.36 ± 0.53<br>↑↑↑		0.70 ± 0.16	2.41 ± 0.41<br>↑↑↑ ▲▲▲	0.97 ± 0.30
P2ry12	0.19 ± 0.16<br>↓↓↓	0.08 ± 0.05<br>↓↓↓	0.17 ± 0.05<br>↓↓↓		0.40 ± 0.09<br>↓	0.42 ± 0.07<br>↓↓↓	0.34 ± 0.06<br>↓↓↓
Sirpa	0.81 ± 0.17	0.72 ± 0.15<br>↓	1.18 ± 0.18<br>▲		0.51 ± 0.03<br>↓↓↓	0.29 ± 0.04<br>↓↓↓ ▼▼▼	0.67 ± 0.06<br>↓↓↓ ▲▲
