# Supplementary material for: Microglia Responses to Pro-inflammatory Stimuli (LPS, IFNγ+TNFα) and Reprogramming by Resolving Cytokines (IL-4, IL-10)
Source: Front Cell Neurosci. 2018 Jul 24;12:215. doi: 10.3389/fncel.2018.00215 (PMC6066613; doi:10.3389/fncel.2018.00215)
Supplement: Supplementary file 8 [file Table_8.pdf]

Microglia responses to pro-inflammatory stimuli (LPS, IFNγ + TNFα) and reprogramming by resolving cytokines (IL-4, IL-10)

Starlee Lively and Lyanne C. Schlichter*

* Correspondence: Professor Lyanne C. Schlichter Lyanne.Schlichter@uhnresearch.ca

Supplementary Table 8. Repolarization: K^+ and Ca^{2+} channels and regulators; Ca^{2+}-signaling molecules. Rat microglia were stimulated with LPS or IFNγ + TNFα (I+T) and 2 h later, IL-4 or IL-10 was added for a further 22 h. Results are shown as fold changes (mean ± SD). Arrows indicate statistical differences from unstimulated control cells; while arrowheads show effects of IL-4 or IL-10 on LPS- or I+T-treated cells (decreases in red; increases in blue). n=6–7 individual cultures for every condition. Results were analyzed by 1-way ANOVA (with Tukey's test); one symbol of any type indicates $p<0.05$; two, $p<0.01$; three, $p<0.001$.

Gene	*Fold change with respect to Control* (± SD)					
	LPS	+IL-4	+IL-10	I+T	+IL-4	+IL-10
Calm1 (CaM)	5.96 ± 1.40 ↑↑↑	1.73 ± 0.27 ↑↑↑▼▼▼	3.50 ± 0.62 ↑↑↑▼▼▼	1.75 ± 0.19 ↑↑↑	0.83 ± 0.12 ▼▼▼	1.99 ± 0.29 ↑↑↑
Kcna2 (Kv1.2)	0.63 ± 0.55	0.18 ± 0.13 ↓↓	0.29 ± 0.18	0.12 ± 0.07 ↓↓	0.05 ± 0.03 ↓↓↓	0.13 ± 0.08 ↓↓↓
Kcna3 (Kv1.3)	1.97 ± 0.69 ↑↑	1.81 ± 0.66 ↑	1.57 ± 0.35	2.30 ± 0.47 ↑↑↑	0.91 ± 0.16 ▼▼▼	2.52 ± 0.42 ↑↑↑
Kcna5 (Kv1.5)	6.40 ± 6.62	2.17 ± 1.63	2.73 ± 2.44	1.73 ± 1.10	0.37 ± 0.04 ▼▼	1.05 ± 1.08
Kcnj2 (Kir2.1)	9.04 ± 1.45 ↑↑↑	2.37 ± 0.80 ↑↑↑▼▼▼	7.56 ± 1.13 ↑↑↑	5.13 ± 0.92 ↑↑↑	0.21 ± 0.06 ↓↓↓▼▼▼	6.38 ± 1.75 ↑↑↑
Kcnma1 (BK)	3.24 ± 1.77	2.37 ± 1.45	2.03 ± 0.93	0.84 ± 0.70	0.28 ± 0.04 ↓	1.44 ± 0.60
Kcnn3 (KCa2.3)	11.39 ± 6.35 ↑↑↑	2.33 ± 1.67 ▼▼	7.63 ± 1.80 ↑↑↑	1.47 ± 0.48	0.21 ± 0.10 ↓▼▼▼	1.63 ± 0.33
Kcnn4 (KCa3.1)	1.05 ± 0.96	0.66 ± 0.21	0.85 ± 0.33	1.99 ± 0.45	0.86 ± 0.38 ▼▼▼	2.72 ± 0.68 ↑↑↑
Mtmr6	3.57 ± 0.62 ↑↑↑	1.33 ± 0.18 ↑▼▼▼	2.63 ± 0.32 ↑↑↑▼	1.56 ± 0.16 ↑↑↑	0.70 ± 0.06 ↓↓▼▼▼	1.63 ± 0.33 ↑↑↑
Nme2 (NDPK-B)	2.90 ± 0.25 ↑↑↑	3.17 ± 0.19 ↑↑↑	2.25 ± 0.32 ↑↑↑▼▼	0.87 ± 0.11	1.43 ± 0.11 ↑↑↑▲▲▲	0.98 ± 0.08
Orai1	4.56 ± 0.76 ↑↑↑	2.59 ± 0.60 ↑↑↑▼▼▼	4.25 ± 0.98 ↑↑↑	1.06 ± 0.11	0.81 ± 0.07 ▼	1.32 ± 0.24 ↑
Orai3	3.59 ± 0.79 ↑↑↑	1.29 ± 0.27 ▼▼▼	2.59 ± 0.40 ↑↑↑▼	2.22 ± 0.11 ↑↑↑	0.76 ± 0.05 ↓↓▼▼▼	2.63 ± 0.48 ↑↑↑
Phtp1	2.28 ± 0.41 ↑↑↑	1.42 ± 0.25 ↑↑▼▼▼	2.05 ± 0.15 ↑↑↑	1.05 ± 0.11	0.83 ± 0.09 ▼	1.18 ± 0.18
Ptpn6 (SHP-1)	1.77 ± 0.27 ↑↑	0.51 ± 0.09 ↓↓↓▼▼▼	2.16 ± 0.18 ↑↑↑	1.20 ± 0.13	0.89 ± 0.13 ▼▼	1.30 ± 0.13 ↑
Rest	4.40 ± 0.83 ↑↑↑	2.97 ± 0.78 ↑↑↑▼	3.74 ± 0.90 ↑↑↑	3.93 ± 0.37 ↑↑↑	0.79 ± 0.16 ↓▼▼▼	4.54 ± 0.87 ↑↑↑

Slc8a1 (NCX1)	0.42 ± 0.14 ↓↓↓	0.18 ± 0.03 ↓↓↓ ▼▼▼	0.50 ± 0.05 ↓↓↓		0.68 ± 0.06 ↓	0.26 ± 0.02 ↓↓↓ ▼▼▼	0.72 ± 0.08 ↓↓↓
Stim1	2.04 ± 0.56 ↑↑↑	0.46 ± 0.13 ↓↓↓ ▼▼▼	1.62 ± 0.22 ↓↓		2.23 ± 0.25 ↑↑↑	0.79 ± 0.06 ↓ ▼▼▼	2.27 ± 0.36 ↑↑↑
Stim2	1.57 ± 0.43 ↑↑	0.57 ± 0.09 ↓↓↓ ▼▼▼	1.03 ± 0.18 ▼▼		1.25 ± 0.17	0.64 ± 0.08 ↓↓↓ ▼▼▼	1.16 ± 0.20
Trpm2	1.14 ± 0.28	0.63 ± 0.13	1.59 ± 0.20		1.73 ± 0.42	1.04 ± 0.18	2.14 ± 0.55 ↑↑
Trpm4	3.43 ± 1.31 ↑↑↑	3.43 ± 1.40 ↑↑↑	3.08 ± 0.88 ↑↑↑		1.15 ± 0.26	0.15 ± 0.07 ↓↓↓ ▼▼▼	1.29 ± 0.17
Trpm7	2.54 ± 0.36 ↑↑↑	1.39 ± 0.42 ▼▼▼	2.08 ± 0.35 ↑↑↑		1.18 ± 0.06 ↑	0.57 ± 0.04 ↓↓↓ ▼▼▼	1.39 ± 0.14 ↑↑↑ ▲▲
