# Supplementary material for: Microglia Responses to Pro-inflammatory Stimuli (LPS, IFNγ+TNFα) and Reprogramming by Resolving Cytokines (IL-4, IL-10)
Source: Front Cell Neurosci. 2018 Jul 24;12:215. doi: 10.3389/fncel.2018.00215 (PMC6066613; doi:10.3389/fncel.2018.00215)
Supplement: Supplementary file 9 [file Image_1.pdf]

Microglia responses to pro-inflammatory stimuli (LPS, IFNγ + TNFα) and reprogramming by resolving cytokines (IL-4, IL-10)

Starlee Lively and Lyanne C. Schlichter*

*** Correspondence:** Professor Lyanne C. Schlichter Lyanne.Schlichter@uhnresearch.ca

A

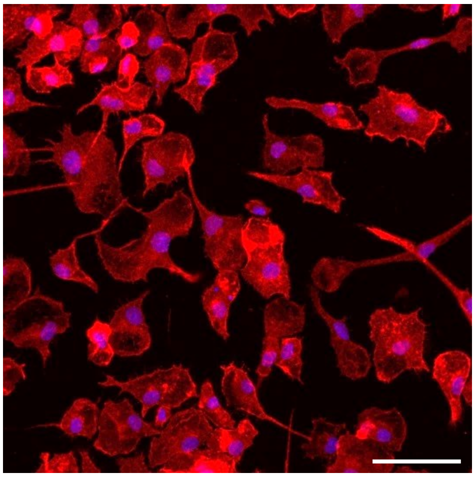

B

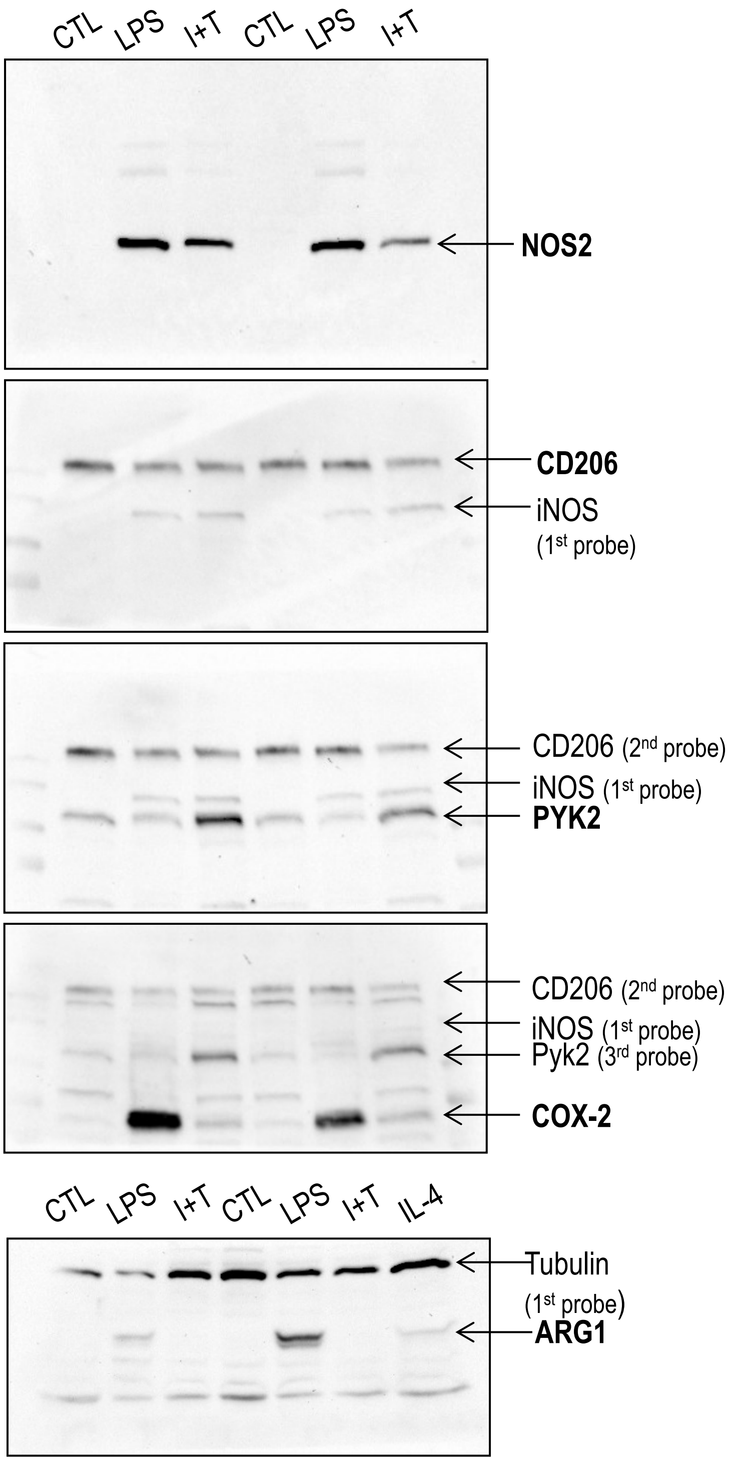

Supplementary Figure 1. A. Cultures were 99 to 100% microglia; i.e., nearly all cells (blue DAPI-stained nuclei) labeled with anti-CD11b antibody (red). Scale bar, 50 µm. B. Examples of uncropped Western Blots used for quantification of single stimulation effects on microglial protein levels. In a pilot study, uncut blots were used to test antibody specificity; however, because samples were limited, blots were cut into strips to probe for proteins of differing weights. When protein weights were similar (e.g., CD206, PYK2, NOS2, COX-2), blots were sequentially reprobed with a different antibody as shown here for NOS2, CD206, PYK2 and COX-2. ARG1 is a different blot and includes rat microglia treated with IL-4 as a positive control.
