# Supplementary material for: Microglia Responses to Pro-inflammatory Stimuli (LPS, IFNγ+TNFα) and Reprogramming by Resolving Cytokines (IL-4, IL-10)
Source: Front Cell Neurosci. 2018 Jul 24;12:215. doi: 10.3389/fncel.2018.00215 (PMC6066613; doi:10.3389/fncel.2018.00215)
Supplement: Supplementary file 10 [file Image_2.pdf]

Microglia responses to pro-inflammatory stimuli (LPS, IFNγ + TNFα) and reprogramming by resolving cytokines (IL-4, IL-10)

Starlee Lively and Lyanne C. Schlichter*

* **Correspondence:** Professor Lyanne C. Schlichter Lyanne.Schlichter@uhnresearch.ca

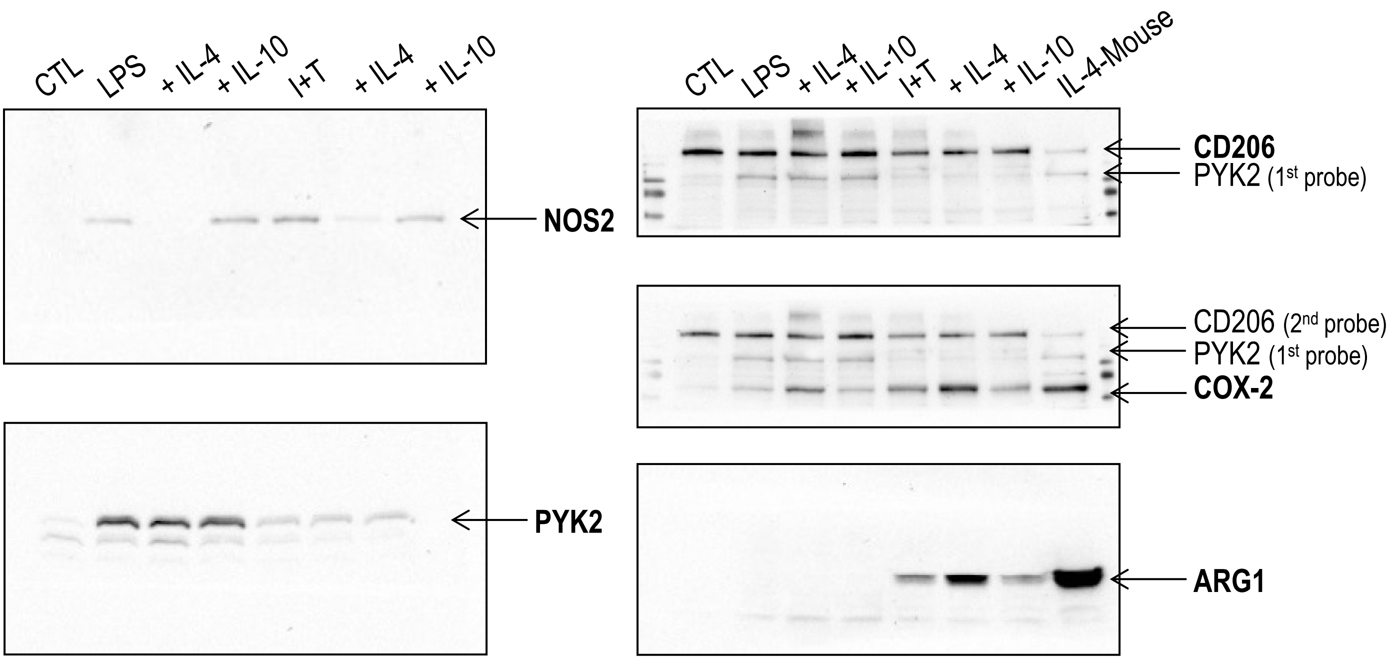

Supplementary Figure 2. Examples of uncropped Western Blots used for quantification of sequential stimulation on microglial protein levels. In a pilot study, uncut blots were used to test antibody specificity; however, because samples were limited, blots were cut into strips to probe for proteins of differing weights. When protein weights were similar (e.g., CD206, PYK2, NOS2, COX-2), blots were sequentially reprobed with a different antibody as shown here for CD206 and COX-2. For some trials, IL-4-treated mouse microglia were included as a positive control.
